# Modeling evolution of spatially distributed bacterial communities: a simulation with the haploid evolutionary constructor

**DOI:** 10.1186/1471-2148-15-S1-S3

**Published:** 2015-02-02

**Authors:** Alexandra Igorevna Klimenko, Yury Georgievich Matushkin, Nikolay Alexandrovich Kolchanov, Sergey Alexandrovich Lashin

**Affiliations:** 1 Institute of Cytology and Genetics SB RAS, Lavrentiev Avenue 10, Novosibirsk, 630090, Russia; 2 Novosibirsk State University, Pirogova st. 2, Novosibirsk 630090, Russia

**Keywords:** Microbial community, spatial distribution, evolutionary modeling, prokaryotes

## Abstract

**Background:**

Multiscale approaches for integrating submodels of various levels of biological organization into a single model became the major tool of systems biology. In this paper, we have constructed and simulated a set of multiscale models of spatially distributed microbial communities and study an influence of unevenly distributed environmental factors on the genetic diversity and evolution of the community members.

**Results:**

Haploid Evolutionary Constructor software http://evol-constructor.bionet.nsc.ru/ was expanded by adding the tool for the spatial modeling of a microbial community (1D, 2D and 3D versions). A set of the models of spatially distributed communities was built to demonstrate that the spatial distribution of cells affects both intensity of selection and evolution rate.

**Conclusion:**

In spatially heterogeneous communities, the change in the direction of the environmental flow might be reflected in local irregular population dynamics, while the genetic structure of populations (frequencies of the alleles) remains stable. Furthermore, in spatially heterogeneous communities, the chemotaxis might dramatically affect the evolution of community members.

## Background

Prokaryotes considered as the most ancient living organisms and essential part of the Earth biosphere. In particular, prokaryotic (or microbial) communities maintain all major biogeochemical cycles [1-3]. Typical examples of the communities are spatially complex, layered structures of the bacterial mats or biofilms [4-6]). A majority of prokaryote species cannot be cultured, so we have to study them within their natural environment, i.e. in communities. Hence, mathematical modeling and simulation of bacterial communities are indispensable for understanding of the functioning and evolution of bacteria.

Spatial factors are well-known to be among major forces of the evolution [7-11]. From ecological and evolutionary points of view, spatial distribution of species plays a large role in local microbial cooperation and competition; consequently, spatial distribution influences evolution [4,12-15]. Combined with other evolutionary factors, these factors affect dynamics of allele frequencies in populations of a community [16-20]. Thus, it has been shown that mutator populations adapted faster than wild-type populations in both liquid and solid environments. Also, it has been shown that independently of the mutation rate, the increase in fitness in the spatially structured environment was smaller than in the unstructured one [21]. Summarizing the mentioned above, the study of dependence of functioning and evolution of microbial communities on the spatial distribution of organisms and substances is of essential theoretical interest. Equally important is the study of how various communities transform their habitats.

At present, a number of software tools are available for modeling and simulation of spatially distributed microbial communities. A majority of these tools, such as cellular automata UMCCA [22], multi-agent software packages AgentCell [23], AQUASIM [24], INDISIM [25] and others [26-30], emphasize the details of the spatial distribution of cells as such. However, the study of the genetic variability effects upon a spatial structure of a community is of equal importance. On the other hand, evolutionary oriented software packages, for example, AEvol [31,32] and others [33-35], mainly focus on genetic structure, evolution and/or metabolism and do not provide the modules for the study of spatial organization. In present study, we took into account both the spatial distribution of cells and substances and the genetic variability of individual populations by developing a software with expanded set of options that permits the requested modeling.

In this paper, we have carried out the modeling of the spatial heterogeneity of environment surrounding the microbial community and its influence on population dynamics and evolution. For this purpose, we have developed a software package for the modeling of spatially distributed microbial communities (1D, 2D, and 3D versions); the package was added to Haploid Evolutionary Constructor (HEC) software that was described previously [36]. We have constructed and analyzed computational models of the prokaryotic communities' evolution with "poisoner-prey" trophic interactions in heterogeneous flow environments. We have also studied models of the horizontal gene transfer in prokaryotic communities living in spatially distributed habitats under changing environmental conditions. According to our models, a combination of chemotaxis with other spatial factors might significantly affect the life of prokaryotic communities through the changes in both the dynamics of population and its genetic structure.

## Modeling methods

**Figure 1:**
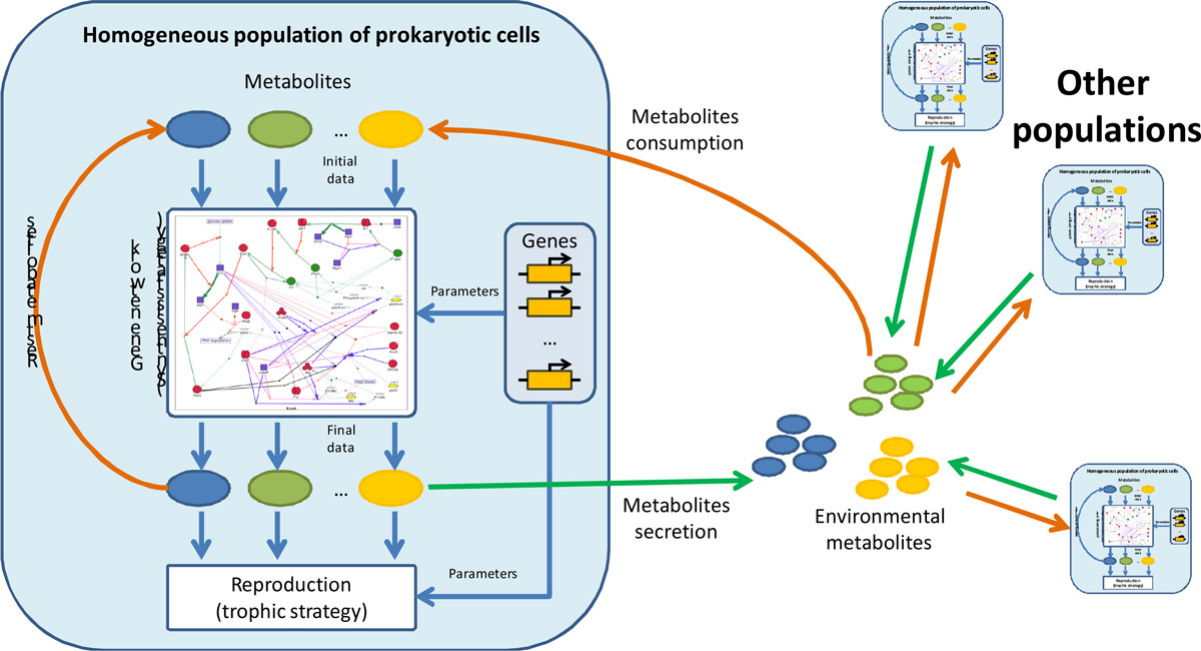
**Principal diagram of main HEC objects and processes in the 0D case (uniform mixing **[36]).

Simulations have been carried out with the HEC software. The key object modeled in the HEC is the prokaryotic *polymorphic population *which is assumed here to be equivalent to species (or strain). Populations consist of cells utilizing substrates which are either consumed from the environment or are synthesized by themselves. Utilization energy is then used for the reproduction and synthesis of other substrates. Synthesized substrates may be either used by cells for own requirements or secreted into habitat. In the latter case, substrates may be consumed by cells of other species. In the HEC, substrate synthesis and utilization, as well as cell reproduction, are described via the corresponding gene networks (GN) which are named *strategies *(i.e. there are synthesis strategies and reproduction strategies, see details in Figure 1). Numerical parameters of those GNs assumed to be "genes" inheriting across the generations. Cells are supposed to belong to the same population (species) if they possess the same GN structures. However, numerical parameters of GNs in cells of a certain population may vary that models the genetic polymorphism. The *generalized genome *of a polymorphic population is formed by a set of allele distributions for each "gene". Mutations changing numerical values of GN parameters consequently change the corresponding allele distribution. Mutations may either be pre-described by the user, or be randomly generated with beta distribution (the user may also control its parameters). In the HEC, both finite and infinite sites models may be used optionally. At the same time, horizontal gene transfer or genes changes the structure of GN, which, in turn, aids in generation of novel species. These processes may also be pre-described by the user or be randomly generated. Thus, the polymorphic population is characterized by the generalized population genome, the synthesis strategy, the reproduction strategy, intercellular substrates and other parameters (Additional file 1 fig. S1, detailed description is published in [37,38]). Most of the HEC parameters concerning the cell size, amounts of substrates required for cell reproduction and other factors have been estimated on the base of *E.coli *data [39]. The simulations describe the dynamics of populations, dynamics of allele frequencies (due to either selection or mutations), origin and extinction of species, and dynamics of environmental substrates. All the dynamics of the system is conditioned by the efficiency of metabolic processes of community members in particular environmental conditions (which in turn depend on corresponding GN). Number of classical models including the logistic growth [40] of the bacterial population and Fisher's fundamental theorem of natural selection [41,42] had previously been implemented in the HEC and found to be consistent with the model expectations [36].

**Figure 2:**
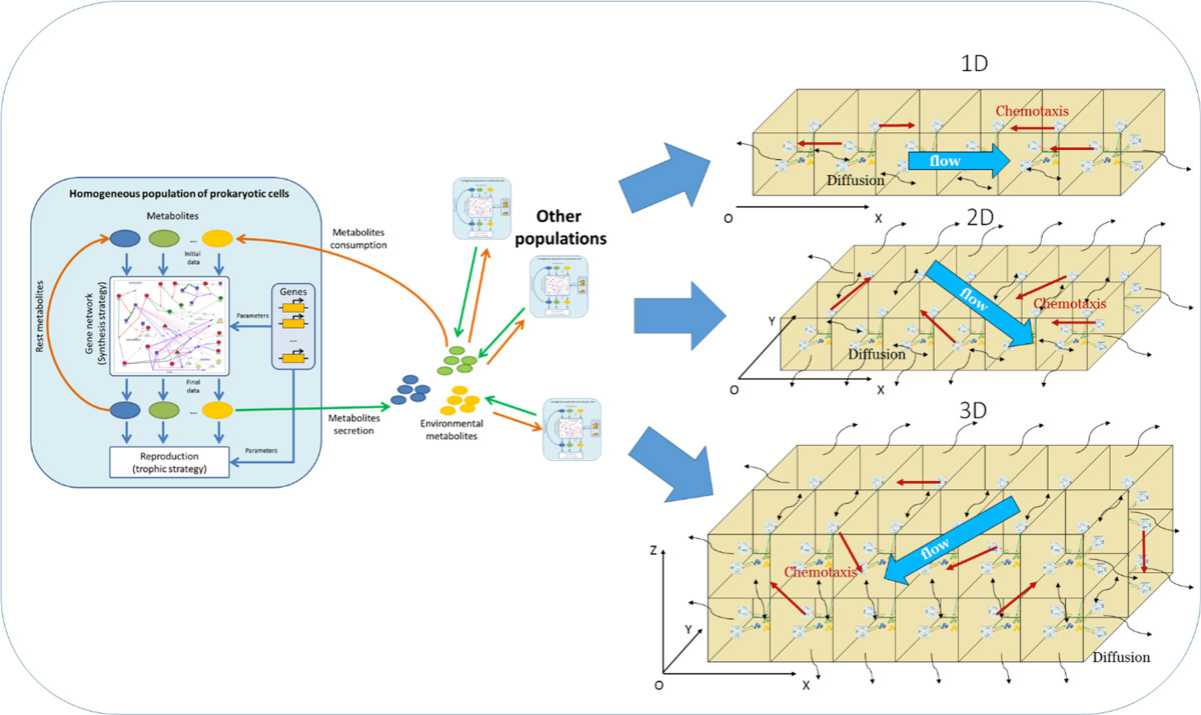
**Building 1D, 2D and 3D environments form 0D blocks**.

We have extended the original HEC (uniform mixing case, denoted as 0D [36]) by adding the cases of one-, two- or three-dimensional spatial distributions of cells and substrates into the model (1D, 2D and 3D versions of the HEC, correspondingly). We use the grid of "point environments" - finite mesh of little volume connected with continuous flow (Figure 2) to model spatial distributions in HEC 1D-3D.

Standard simulation step in the HEC 1D-3D consists of the following two stages:

(1) calculation of new states for each point environments (which is independent and can be performed simultaneously) including simulation of the following processes: consumption of substrates, utilization of substrates, reproduction, substrate synthesis and secretion. This stage is apparently inherited from the HEC 0D;

(2) spatial redistribution of substrates and cells in the modeled system including the simulation of flow, diffusion, and chemotaxis processes (Figure 2). Detailed description of spatial processes, as well as nuances related to differences in characteristic times of reproduction and spatial processes, is presented in Additional file 1 (fig. S3, S4).

Hence, the processes of substrate production and utilization, reproduction, mutation, genes loss and horizontal transfer are simulated as part of the standard HEC 0D iteration and occur independently in the each mesh point (Additional file 1 fig. S2). Only spatial redistribution of organisms and substrates requires nodes synchronization (Additional file 1 fig. S3).

## Results and discussion

### Modeling "poisoner-prey" community in spatially heterogeneous habitats

**Figure 3:**
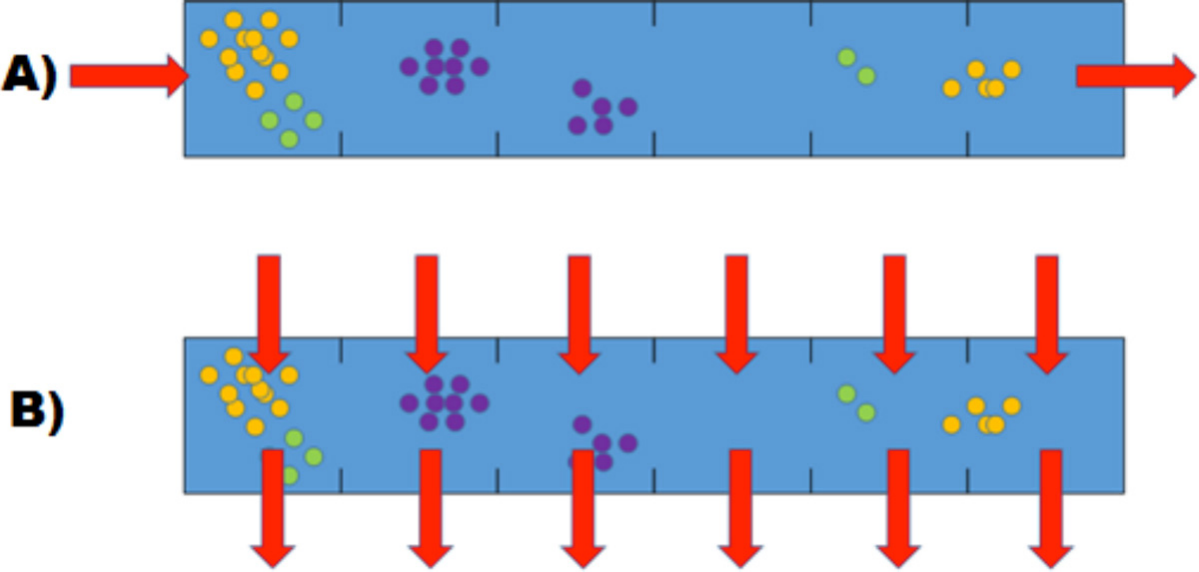
**Spatial organization of a habitat: a) flow-through; b) perpendicular-flow**.

**Figure 4:**
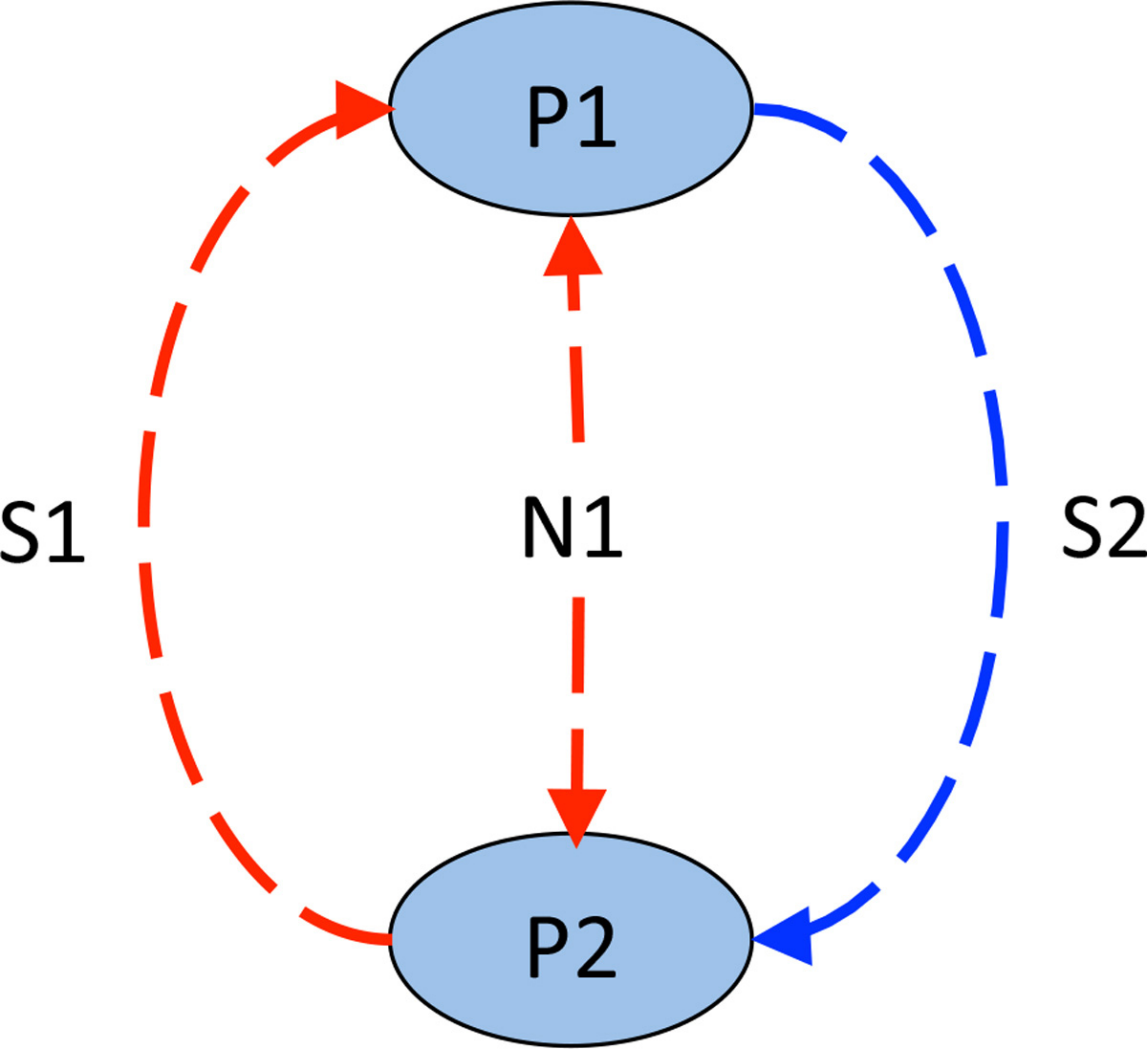
**Trophic graph of the "poisoner-prey" community**. P1 - poisoner population, P2 - prey population, S1 - substrate synthesized by the prey, S2 - substrate (toxin) synthesized by the poisoner, N1 - non-specific substrate coming with the inflow.

Using the methods described above, we have studied the change of genetic diversity in populations of the "poisoner-prey" community. The "poisoner-prey" model has been previously described in our recent studies [36,43]. One-dimensional flow-through environment (1D tube, Figure 3). Microbial community consisted of two populations - the poisoner and the prey (Figure 4). Metabolic by-product secreted by the poisoners would inhibit the growth of the prey which product would conversely activate the growth of the poisoner population. Besides, both populations would consume non-specific substrate which had come into the habitat with the inflow. They used it for the growth. In this model (as well as in the others in this paper) we have considered systems of various nodes number (from 10 to 1000). Since a qualitative character of model behavior remained the same, we have considered 10-node tubes without losing generality in this paper. Furthermore, as we have not simulated any mutation, the allele frequencies changed only by the efficiency of metabolic processes of community members in particular environment, which, in turn, depends on corresponding GN). For each allelic combination in a polymorphic population P, the fitness is calculated as the (P_after_-P_before_)/P_before _ratio, where P_before _and P_after _denote the size of P before and after the reproduction process, respectively.

In the described model, we have considered the following ways of a spatial organization:

1. Flow-through habitat (Figure 3a);

2. Perpendicular-flow habitats (Figure 3b).

First, we have considered a model (Additional file 2) with the uniform initial distribution of cells and substances, and flow-through (Figure 3a) spatial organization with the flow rate of 0.02 (i.e. 2% of cells and substances of the node is taken away at each iteration). Initial populations were genetically polymorphic: prey cells varied in terms of their sensitivity to the S2 inhibitor, poisoner cells varied in terms of their efficiency of the S1 substrate utilization. Population dynamics of the community demonstrates (Additional file 1 Fig. S6) that after a relatively short period of oscillation in the preys' size which is associated with the steady growth of the poisoners, the size of both populations becomes stable.

**Figure 5:**
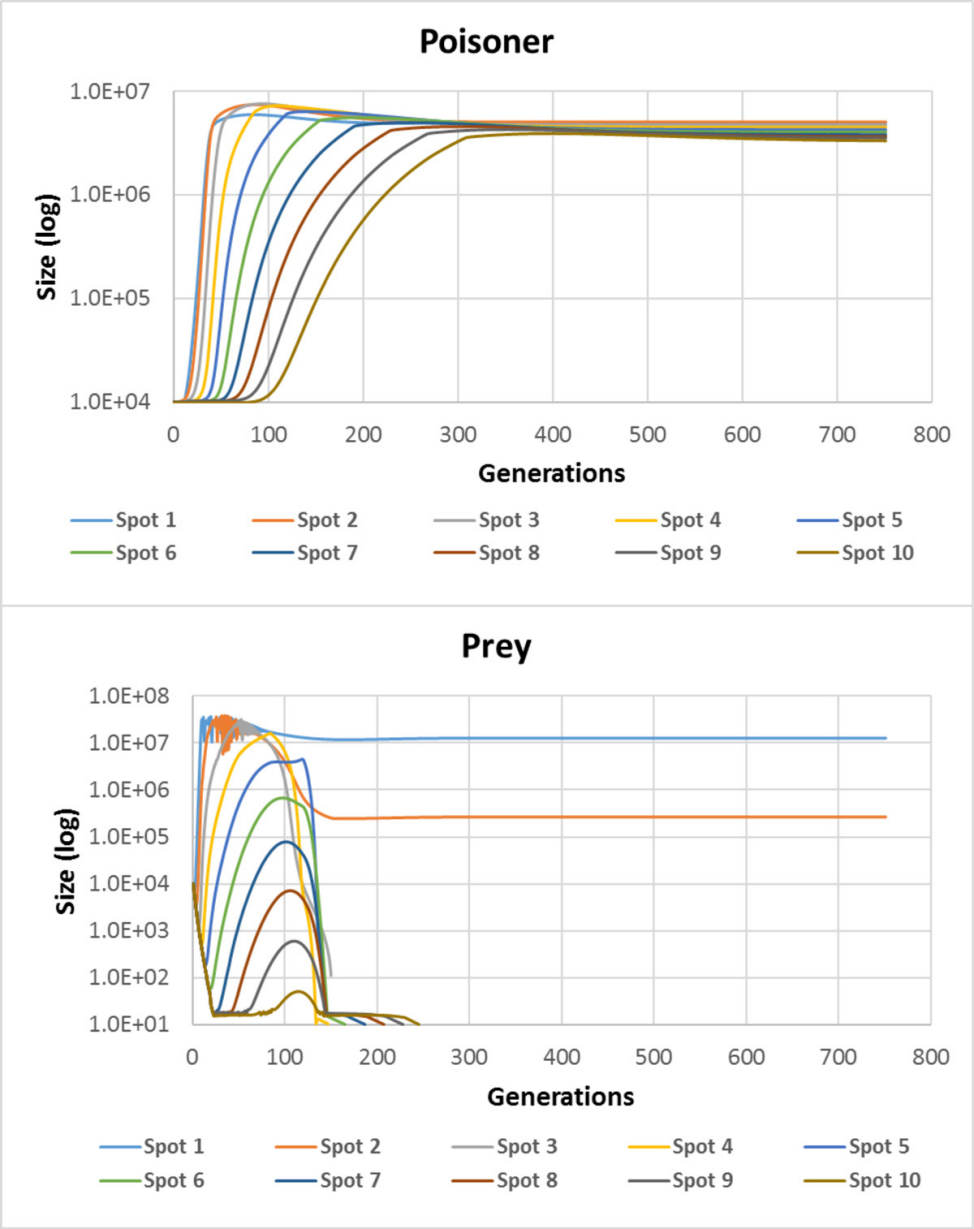
**Population dynamics of the prey (top) and poisoners (bottom) in the "poisoner-prey" model with the initial genetic polymorphism in both populations (Additional file **1). Various colors show various nodes of the habitat.

However, if we look at separate nodes (Figure 5), we can see that after 250 generations the prey survives only in 1^st ^and 2^nd ^nodes, while the poisoner lives in all nodes of the habitat. We think that this is due to the preys which live only on the non-specific substrate which is in sufficient amount only in the nodes close to the inflow. At the same time, the poisoner can partially compensate the lack of the non-specific substrate with the S1.

**Figure 6:**
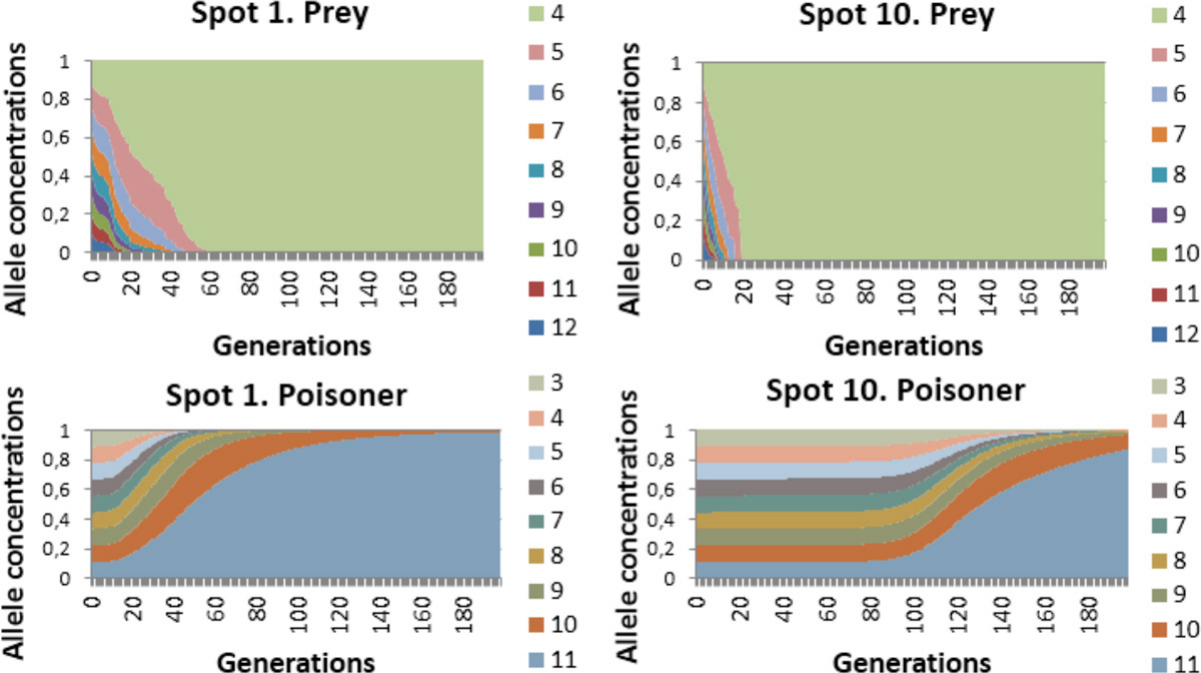
**Dynamics of allele frequencies in prey and poisoner populations in nodes 1 and 10 in the "poisoner-prey" model with the initial genetic polymorphism (Additional file**1). Color width denotes proportion of allele in a population.

At the same time, we have analyzed the change of genetic diversity in both populations. Figure 6 shows that adaptive alleles (i.e. in case with the prey, these are parameters of sensitivity to S2, in case with the poisoner, these are efficiencies of S1 utilization) completely displace less adaptive ones in course of time. Moreover, the nodes outlying from the source of the non-specific substrate are characterized by the rapid genetic diversity loss for the prey population which is associated with the preservation of genetic diversity in the poisoner (Table 1 node 10 in Figure 6). Reverse situation is observed in proximal nodes (Table 1 node 1 in Figure 6).

**Table 1:** Time of genetic diversity loss (extinction of all inadaptive alleles) for the poisoner and prey populations in different nodes.

Poisoner		Prey	
	**Time of genetic diversity loss, generation**		**Time of genetic diversity loss, generation**

**Node 1**	550 ± 2.8	**Node 1**	89 ± 0.2

**Node 2**	551 ± 2.8	**Node 2**	89 ± 0.3

**Node 5**	561 ± 2.9	**Node 5**	104 ± 0.3

**Node 8**	574 ± 3.2	**Node 8**	19 ± 0

**Node 10**	582 ± 2.9	**Node 10**	19 ± 0

One hundred simulation runs have been performed in which the initial allele distributions varied in such a way that the frequency for each allele lied in the range 5-15% (total number of alleles in a distribution was 9). Confidence intervals are stated at the 95% confidence level.

However, in central nodes (node 5 in Table 1) we have observed an increase of time of the genetic diversity preservation - inadaptive alleles would extinct only at 104 ± 0.3 generation. We think this can be explained by the flow transferring migrants from proximal regions of a habitat in which relatively high genetic diversity remains.

Therefore, spatial localization of microorganisms may influence the evolutionary rate: depending on the cell position relative to the source of non-specific substrate, we have observed evolutionary rates among poisoners and preys to be differed from each other. Poisoners evolve more rapidly than preys when located near the source of non-specific substrate and vice versa. Located far from the source, the preys evolve more rapidly.

**Figure 7:**
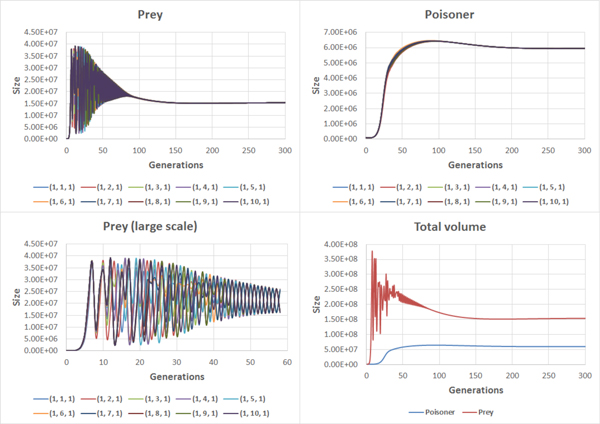
**Population dynamics of preys (left) and poisoners (right) in different nodes and a whole habitat (chemotaxis is off, Additional file**2).

Later we have studied the above model by adding a non-uniform initial state and chemotaxis. Spatial structure of a habitat has been set to perpendicular-flow (Figure 3b). Initial distribution of the inhibitor S2 has been set to gradient (the highest concentration was in the node 1, the lowest in the node 10). Flow rate was the same as in the previous case. We have analyzed the influence of chemotaxis on the model behavior. Figure 7 shows the chemotaxis-off case (Additional file 3). In different nodes oscillations in the prey population size vary due to non-uniform distribution of inhibitor. Poisoners population size negligibly varies in different nodes.

Situation changes when chemotaxis switches on: cells can actively move to nodes with more favorable conditions. Simulation results are shown in Additional file 1 fig. S7, S8. Inhibitor gradient and cell movement cause an origin of local oscillations in population dynamics which affects the dynamics of both separate nodes and a habitat in whole.

Analysis of this model under various environmental conditions has shown that the non-uniform distribution of substrates that was due to either initial inequalities data or high diffusion rate along with the chemotaxis resulted in emergent community dynamics evident in a movement of cells to a better place of living, into the nodes with higher concentrations of necessary substrates. Under these circumstances, both local oscillations and even an irregular behavior were observed (Additional file 1 fig. S9). This finding is in an agreement with experimentally observed behavior of antagonistic *E.coli *strains reported earlier [44].

Detailed analysis of the genetic structure dynamics in these populations is described below. We have considered the "poisoner-prey" model with a perpendicular flow and a diffusion-dependent non-uniformity of substrates. At the baseline, the genetic polymorphism was same to that in previous models (Figure 6). Population dynamics of the model is shown in Additional file 1 fig. S10.

Diffusion causes non-uniformity. Combined with active movement of cells, it leads to irregularities in the dynamics within local populations (Additional file 1 fig. S10). It is evident that chemotaxis may promote irregular oscillations of the population size in separate nodes while at the genetic level the dynamics of allele frequencies remains stable (Additional file 1 fig. S11).

### Modeling horizontal transfer of genes in spatially distributed systems under varying environmental conditions

**Figure 8:**
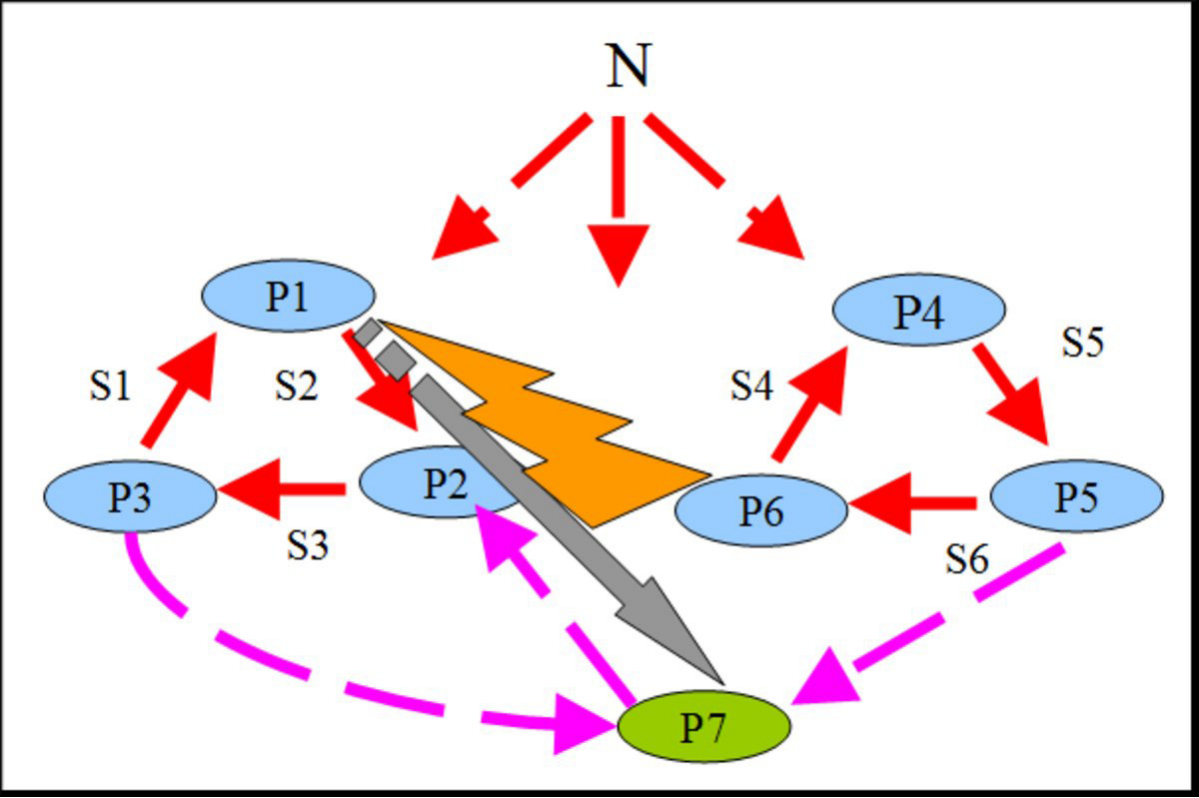
**Trophic graph of a community consisting of two trophic cycles: P1-P2-P3 and P4-P5-P6 **[38]. N is a non-specific substrate contained in the flow. HT of the gene from P6 cells to P1 cells is shown by a lightning. As a result of the HT, the new type of cells, which forms P7 population (grey arrow) originates.

The second model describes co-functioning and competition of populations in a community consisting of two trophic cycles living in the 1D tube habitat (Figure 3) under varying environmental conditions. This model is the extension of a previously published model with an uniform mixing (0D) [38]. Each trophic cycle includes three populations (Figure 8). For this community, we have considered two combinations of breeding strategies: NC-NC and C-C. Here, NC-NC strategy implies that all populations employ non-compensatory strategy, implementing the Liebig's law of the minimum: "The growth is controlled not by the total amount of resources available, but by the most scarce resource (limiting factor)" [45]; C-C means that all populations of a community breed via the compensatory reproduction strategy, implementing the Rubel's law of compensation of ecological factors: "If one factor intensifies the action of another, the minimum of the latter is less than it would be without the help of the former. The contrary effect is possible when the accompanying factor results in raising the minimum" [45] (formulas for both strategies are presented in the Supplementary materials). Our previous studies for the 0D case have shown that during long period of starvation the non-compensatory strategy preserved the genetic diversity while compensatory strategy was preserving it only for a shortwhile [38]. At the same time, non-compensatory systems have been shown to be less adaptable for harsh environmental conditions than compensatory ones.

We have considered both cases with and without chemotaxis. For these models, we have studied how the horizontal transfer (HT) of genes and change of environmental conditions affected the community functioning:

1. On the 100^th ^generation, we have simulated the HT of the gene of the S6 specific substrate utilization from cells of the P6 population into cells of the P1 population. It would lead to the origin of new type cells which then would form the P7 population connecting two trophic cycles.

2. On the 2000^th ^generation, we have simulated the decrease of a non-specific substrate concentration in the flow by factor of 100. Such a "starvation" continued for 1000 generations and then initial conditions had been restored.

**Figure 9:**
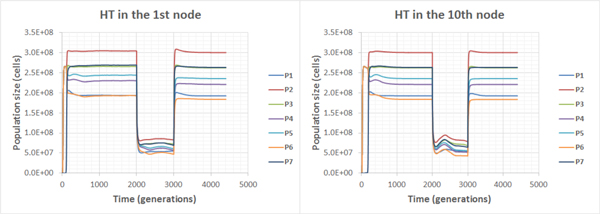
**Population dynamics in the C-C community in the flow-through habitat**. Chemotaxis is on (Additional file 3, Additional file 4).

First, we have considered flow-through habitats. In C-C communities when chemotaxis was switched off, the HT would change the fate of the communities if only occurred in nodes close to the source of a non-specific substrate (nodes 1-4, Additional file 1 fig. S12): new population would survive and population sizes would differentiate. If the HT occurred in outlying nodes (5-10), new population could not settle and would be eliminated soon. Chemotaxis encouraged fixation (Additional file 4 and Additional file 5) and sustaining of biodiversity (surviving of the P7 population). However, total biomass of such a community turned out to be lower (Figure 9).

Palaeontologist Zherikhin and paleobotanist Meyen proposed the concepts of zonal stratification [46] and phytospreading [47], which are the extensions of Darlington's "equatorial pump" concept [48]. According to these concepts, the most rates of taxa formation are observed in such locations in which the struggle for existence against abiotic factors is reduced. In the previous model (chemotaxis off case), the P7 population survived only if the HT occurred in substrate-rich nodes (i.e. close to the node 1), which are analogues of Zherikhin's and Meyen's biotopes. In poor ecosystems, the novel species could not grow up to the necessary size for an effective competition in the community in spite of the fact that novel genotype would be potentially more adaptive than others (more substrates could be utilized). It is in good agreement with the concepts of equatorial pump and phytospreading, as the evolutionary success of a novel species is related to not only pre-adaptations, but also to habitat conditions [49]. There is also an interesting conclusion of the habitat constraint mentioned above. It is commonly supposed that adaptations appear in one or several individuals. If they occur in unfavorable conditions, a small subpopulation of mutants would not fixate in a community owing to its size. That is why pre-adaptations should be accumulated in the form of a neutral variability during the period of relatively optimal habitat conditions. They manifest when conditions change.

**Figure 10:**
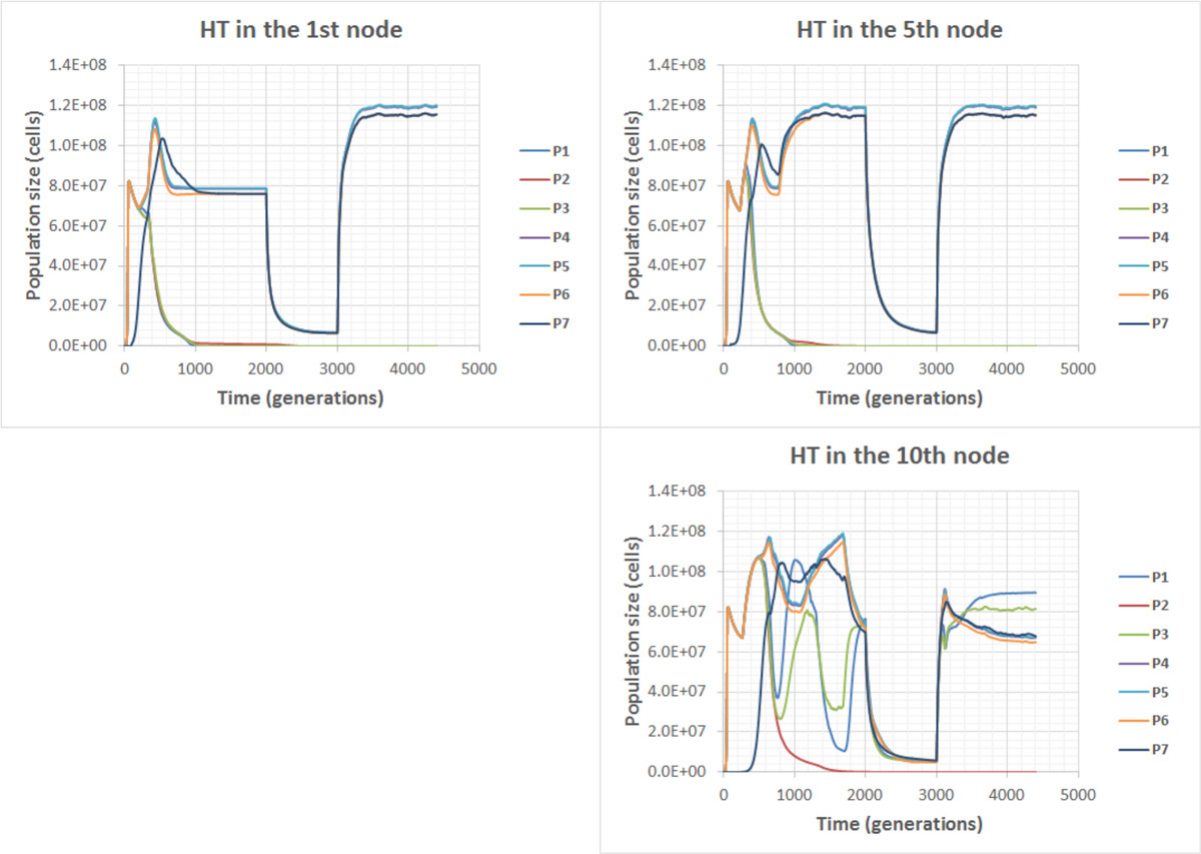
**Population dynamics in the NC-NC community in the flow-through habitat**. Chemotaxis on (Additional file 5 , Additional file 6 , Additional file 7).

**Figure 11:**
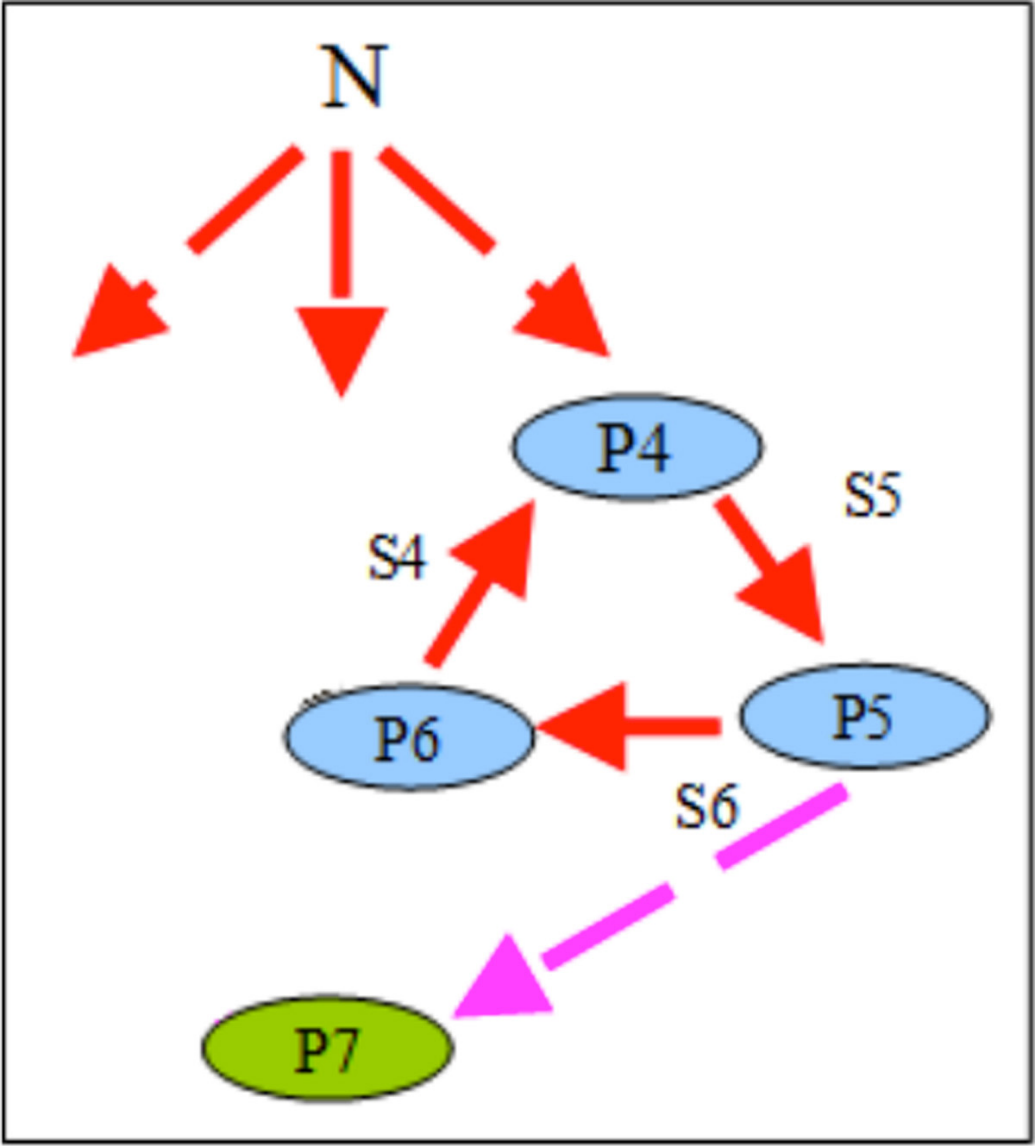
**Graph of trophic interactions in the NC-NC community in the flow-through habitat (chemotaxis is on) after the HT and starvation (HT occurred in 1^st ^- 5^th ^nodes)**.

**Figure 12:**
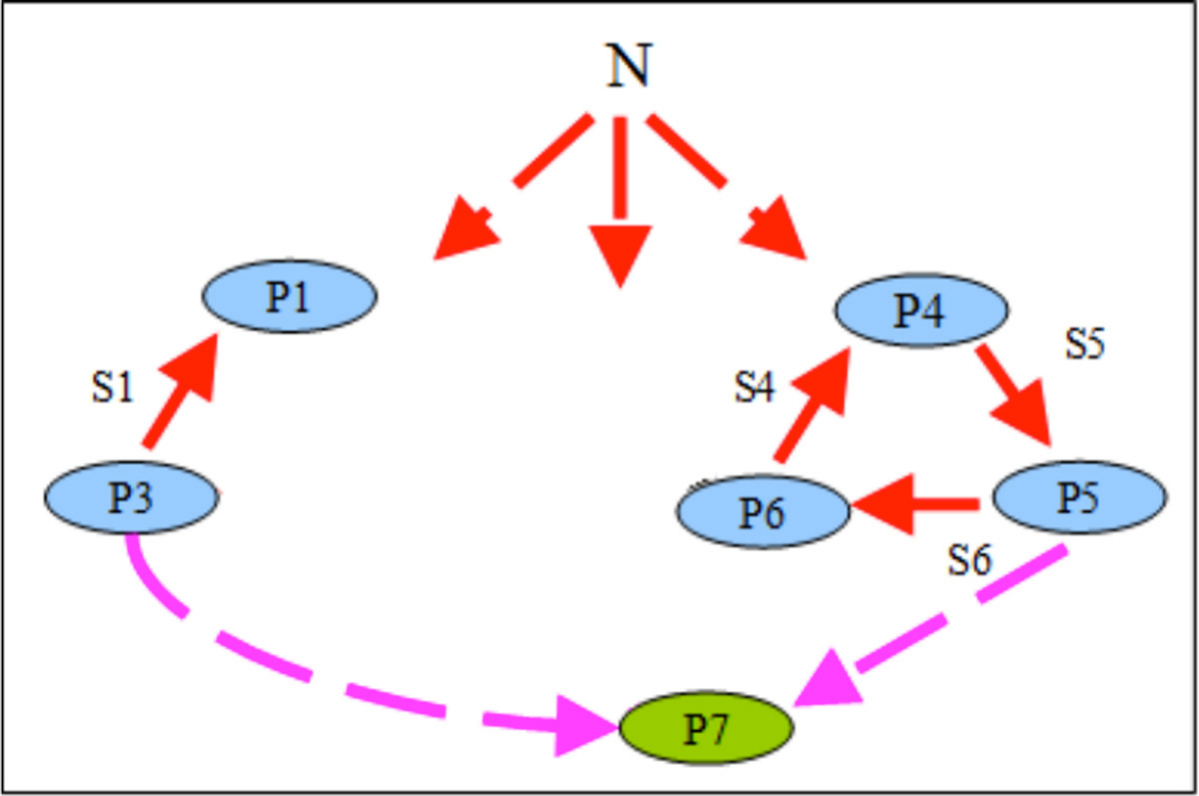
**Graph of trophic interactions in the NC-NC community in the flow-through habitat (chemotaxis is on) after the HT and starvation (HT occurred in the 10^th ^node)**.

Simulation results were similar to those for compensatory communities in non-compensatory communities without chemotaxis. However, in contrast to the C-C case, the presence of chemotaxis has dramatically changed the fate of NC-NC communities. HT has destabilized the system leading to the extinction of the part of the acceptor trophic ring (Figure 10): if the HT occurred close to the source of a non-specific substrate (Additional file 6), the whole acceptor trophic ring would perish (Figure 11); in other cases (Additional file 7 and Additional file 8) only the P2 population would perish (Figure 12).

Finally, we have considered systems with perpendicular-flow habitats. In C-C communities, chemotaxis was found to result in a bit more expressed differentiation of the population size under starvation (Additional file 1 fig. S13) - P_max_/P_min _ratios are 1.77 (chemotaxis is off, Additional file 9 ) and 1.97 (chemotaxis is on, Additional file 10). In NC-NC communities (Additional file 11 and Additional file 12 ), the cell movement ability would lead to the origin of irregular oscillations (Additional file 1 fig. S14), although of a severely limited amplitude (~4*10^7 ^cells).

It seems surprising to us that while the P2 is the potential beneficiary of the community perturbations caused by the HT (initially the P2 cells were fed only by P1 cells, and after HT - by both P1 and P7). It should particularly be noted that the destabilization of the community occurred prior to the starvation. The novel P7 population is rather adaptive as it always survives. However, the P7 does not dominate in the community, which is rather not obvious. In our opinion, the reason is that the NC trophic ring consists of the highly specialized symbiotrophic populations realizing the optimal system of trophic interactions. If the HT along with the chemotaxis promotes the origin of high-competing species and then such species may destabilize the established community even long before the starvation. It consequently may lead to unpredictable dynamics of the community.

The location of the HT significantly affects the fate of communities living in flow-through habitats leading to the differentiation and even the change of the community structure. In perpendicular-flow habitats, the location of the HT plays much lesser role: functional modes of a community do not change whenever the HT occurred.

## Conclusion

In this study, the methods for modeling spatially distributed microbial communities with changing genetic structure have been presented along with the HEC 1D-3D software package. The software allows one to build a model of the microbial community that takes into account both spatial environmental factors and genetic and metabolic variations. Thus, the software described provides it users with expanded simulation capabilities and is superior to existing tools for simulation of microbial communities.

The models of functioning and evolution of prokaryotic communities implementing "poisoner-prey" trophic interactions in spatially heterogeneous habitats have been constructed and analyzed. Spatial localization of organisms has been shown to affect the selection intensity and their evolutionary rate: depending on the distance from a substrate source, we have observed evolutionary rates among poisoner and prey populations to be differed from each other. For "poisoner-prey" communities living in perpendicular-flow habitats, we have also shown that spatial heterogeneity might lead to the origin of irregular population dynamics, but dynamics of genetic structure (allele frequencies) at the time would remain stable.

The horizontal transfer models in symbiotic communities consisted of two trophic cycles and living in spatially distributed habitats under varying environmental conditions have also been analyzed. Location of the HT origin has been noted to essentially influence the fate of the community, especially in flow-through habitats. As for the temporary starvation, various locations of the HT have determined various population structure and even species composition of the community. In perpendicular-flow habitats, place of the HT origin would play much lesser role: functional regimes of the community would not change whenever it occurred.

Therefore, it has been shown that movement factors (chemotaxis) associated with a non-uniform spatial distribution of cells and substances might dramatically affect the life of communities which has been manifested in both population dynamics and dynamics of genetic diversity of populations for all considered models.

## Abbreviations

HEC: Haploid Evolutionary Constructor

GN: gene networks

HT: horizontal transfer

C: compensatory reproduction strategy

NC: non-compensatory reproduction strategy

## Additional information

Additional information can be found in Additional file 13, 14, 15, 16, 17, 18 and 19.

## Competing interests

The authors declare that they have no competing interests.

## Authors' contributions

KAI has implemented spatially distributed versions of the HEC. KAI and LSA have developed and simulated the models. KAI, MYUG, KNA and LSA have analyzed results and performed biological interpretations. LSA has coordinated the writing of the paper.

All authors have read and approved the final manuscript.

## Supplementary Material

Additional file 1 **Archive containing the supplementary figures**. 7-Zip archive containing the supplementary figures S1-S14. Click here for file

Additional file 2 **Archive containing the HEC script and statistic files of pp_spectre_10(through) model**. 7-Zip archive containing text file with the model script and statistic files concerned the results depicted in figures 5-6, S6, table 1. Click here for file

Additional file 3 **Archive containing the HEC script and statistic files of ort_10_d = 0 model**. 7-Zip archive containing text file with the model script and statistic files concerned the results depicted in figure 7. Click here for file

Additional file 4 **Archive containing the HEC script and statistic files of Reissue.Rubel.through.chem = 0.1.hgt1 model**. 7-Zip archive containing text file with the model script and statistic files concerned the results depicted in figure 9 (left side). Click here for file

Additional file 5 **Archive containing the HEC script and statistic files of Reissue.Rubel.through.chem = 0.1.hgt10 model**. 7-Zip archive containing text file with the model script and statistic files concerned the results depicted in figure 9 (right side). Click here for file

Additional file 6 **Archive containing the HEC script and statistic files of Reissue.Liebig.through.chem = 0.1.hgt1 model**. 7-Zip archive containing text file with the model script and statistic files concerned the results depicted in figure 10. Click here for file

Additional file 7 **Archive containing the HEC script and statistic files of Reissue.Liebig.through.chem = 0.1.hgt5 model**. 7-Zip archive containing text file with the model script and statistic files concerned the results depicted in figure 10. Click here for file

Additional file 8 **Archive containing the HEC script and statistic files of Reissue.Liebig.through.chem = 0.1.hgt10 model**. 7-Zip archive containing text file with the model script and statistic files concerned the results depicted in figure 10. Click here for file

Additional file 9 **Archive containing the HEC script and statistic files of Reissue.Rubel.ort model**. 7-Zip archive containing text file with the model script and statistic files concerned the results depicted in figure S13 (left side). Click here for file

Additional file 10 **Archive containing the HEC script and statistic files of Reissue.Rubel.ort .chem = 0.1 model**. 7-Zip archive containing text file with the model script and statistic files concerned the results depicted in figure S13 (right side). Click here for file

Additional file 11 **Archive containing the HEC script and statistic files of Reissue.Liebig.ort model**. 7-Zip archive containing text file with the model script and statistic files concerned the results depicted in figure S14 (left side). Click here for file

Additional file 12 **Archive containing the HEC script and statistic files of Reissue.Liebig.ort .chem = 0.1 model**. 7-Zip archive containing text file with the model script and statistic files concerned the results depicted in figure S14 (right side). Click here for file

Additional file 13 Simulation of spatially distributed habitats; Breeding strategies formulas Click here for file

Additional file 14 **Archive containing the HEC executable file**. 7-Zip archive containing the HEC executable file (Windows version). Click here for file

Additional file 15 **Archive containing the HEC script and statistic files of ort_10_d = 0 model**. 7-Zip archive containing text file with the model script and statistic files concerned the results depicted in figures S7-S8, S9a. Click here for file

Additional file 16 **Archive containing the HEC script and statistic files of ort_10_d = 0.01 model**. 7-Zip archive containing text file with the model script and statistic files concerned the results depicted in figure S9b. Click here for file

Additional file 17 **Archive containing the HEC script and statistic files of ort_10_d = 0.01(homog) model**. 7-Zip archive containing text file with the model script and statistic files concerned the results depicted in figure S9c. Click here for file

Additional file 18 **Archive containing the HEC script and statistic files of pp_spectre_10(ort) model**. 7-Zip archive containing text file with the model script and statistic files concerned the results depicted in figure S10-S11. Click here for file

Additional file 19 **Archive containing the HEC script and statistic files of Reissue.Rubel.through model**. 7-Zip archive containing text file with the model script and statistic files concerned the results depicted in figure S12. Click here for file

## Declarations

Publication of this article has been funded by the RSF N 14-24-00123 grant.

This article has been published as part of *BMC Evolutionary Biology *Volume 15 Supplement 1, 2015: Selected articles from the IX International Conference on the Bioinformatics of Genome Regulation and Structure\Systems Biology (BGRS\SB-2014): Evolutionary Biology. The full contents of the supplement are available online at http://www.biomedcentral.com/bmcevolbiol/supplements/15/S1.

## References

[B1] Zavarzin G Jørgensen SE Microbial Cycles Glob Ecol 2010 1 Academic Press 183 190

[B2] Dobretsov N Kolchanov N Rozanov A Zavarzin G Biosphere Origin and Evolution 2008 Boston, MA: Springer US 426

[B3] Madigan MT Martinko JM Bender KS Buckley DH Stahl DA Brock Biology of Microorganisms 2014 14 New York: Benjamin Cummings 1136

[B4] Stoodley P Sauer K Davies DG Costerton JW Biofilms as complex differentiated communities Annu Rev Microbiol 2002 56 187 209 10.1146/annurev.micro.56.012302.160705 12142477

[B5] Conrad JC Physics of bacterial near-surface motility using flagella and type IV pili: implications for biofilm formation Res Microbiol 2012 163 619 29 10.1016/j.resmic.2012.10.016 23103335

[B6] Webb JS Givskov M Kjelleberg S Bacterial biofilms: prokaryotic adventures in multicellularity Curr Opin Microbiol 2003 6 578 585 10.1016/j.mib.2003.10.014 14662353

[B7] Hanski I Gaggiotti O Ecology, Genetics and Evolution of Metapopulations 2004 Academic Press 696

[B8] Rousset F Balding DJ, Bishop M, Cannings C Inferences from Spatial Population Genetics Handb Stat Genet Third Ed 2004 Chichester: John Wiley & Sons, Ltd

[B9] Manel S Schwartz MK Luikart G Taberlet P Landscape genetics: combining landscape ecology and population genetics Trends Ecol Evol 2003 18 189 197 10.1016/S0169-5347(03)00008-9

[B10] François O Durand E Spatially explicit Bayesian clustering models in population genetics Mol Ecol Resour 2010 10 773 84 10.1111/j.1755-0998.2010.02868.x 21565089

[B11] Pigolotti S Benzi R Perlekar P Jensen MH Toschi F Nelson DR Growth, competition and cooperation in spatial population genetics Theor Popul Biol 2013 84 72 86 23298763 10.1016/j.tpb.2012.12.002

[B12] Kreft J-U Biofilms promote altruism Microbiology 2004 150 Pt 8 2751 60 15289571 10.1099/mic.0.26829-0

[B13] Xavier JB Foster KR Cooperation and conflict in microbial biofilms Proc Natl Acad Sci USA 2007 104 876 81 10.1073/pnas.0607651104 17210916 PMC1783407

[B14] Kreft J Picioreanu C Wimpenny JWT Van Loosdrecht MCM Individual-based modelling of biofilms Microbiology 2001 147 2897 2912 11700341 10.1099/00221287-147-11-2897

[B15] Holmes AB Kalvala S Whitworth DE Spatial simulations of myxobacterial development PLoS Comput Biol 2010 6 e1000686 10.1371/journal.pcbi.1000686 20195493 PMC2829040

[B16] Rainey PB Travisano M Adaptive radiation in a heterogeneous environment Nature 1998 394 69 72 10.1038/27900 9665128

[B17] Rainey PB Rainey K Evolution of cooperation and conflict in experimental bacterial populations Nature 2003 425 72 4 10.1038/nature01906 12955142

[B18] Klitgord N Segrè D Environments that induce synthetic microbial ecosystems PLoS Comput Biol 2010 6 e1001002 10.1371/journal.pcbi.1001002 21124952 PMC2987903

[B19] Mitri S Xavier JB Foster KR Social evolution in multispecies biofilms Proc Natl Acad Sci USA 2011 108 Suppl 10839 46 21690380 10.1073/pnas.1100292108 PMC3131810

[B20] Mitri S Foster KR The genotypic view of social interactions in microbial communities Annu Rev Genet 2013 47 247 73 10.1146/annurev-genet-111212-133307 24016192

[B21] Perfeito L Pereira MI Campos PRA Gordo I The effect of spatial structure on adaptation in Escherichia coli Biol Lett 2008 4 57 9 10.1098/rsbl.2007.0481 18029298 PMC2412928

[B22] Laspidou CS Rittmann BE Evaluating trends in biofilm density using the UMCCA model Water Res 2004 38 3362 72 10.1016/j.watres.2004.04.051 15276753

[B23] Emonet T Macal CM North MJ Wickersham CE Cluzel P AgentCell: a digital single-cell assay for bacterial chemotaxis Bioinformatics 2005 21 2714 21 10.1093/bioinformatics/bti391 15774553

[B24] Wanner O Morgenroth E Biofilm modeling with AQUASIM Water Sci Technol 2004 49 137 44 15303734

[B25] Ginovart M López D Valls J INDISIM, an individual-based discrete simulation model to study bacterial cultures J Theor Biol 2002 214 305 19 10.1006/jtbi.2001.2466 11812180

[B26] Xavier JB Picioreanu C van Loosdrecht MCM A framework for multidimensional modelling of activity and structure of multispecies biofilms Environ Microbiol 2005 7 1085 103 10.1111/j.1462-2920.2005.00787.x 16011747

[B27] Ferrer J Prats C López D Individual-based modelling: an essential tool for microbiology J Biol Phys 2008 34 19 37 10.1007/s10867-008-9082-3 19669490 PMC2577750

[B28] Tao Y Slater GW A Simulation Model of Biofilms with Autonomous Cells, 2 - Explicit Representation of the Extracellular Polymeric Substance Macromol Theory Simulations 2011 20 571 583 10.1002/mats.201100030

[B29] Resat H Bailey V McCue LA Konopka A Modeling microbial dynamics in heterogeneous environments: growth on soil carbon sources Microb Ecol 2012 63 883 97 10.1007/s00248-011-9965-x 22193925

[B30] Hellweger FL Bucci V A bunch of tiny individuals--Individual-based modeling for microbes Ecol Modell 2009 220 8 22 10.1016/j.ecolmodel.2008.09.004

[B31] Beslon G Parsons DP Sanchez-Dehesa Y Peña J-M Knibbe C Scaling laws in bacterial genomes: a side-effect of selection of mutational robustness? Biosystems 2010 102 32 40 10.1016/j.biosystems.2010.07.009 20655979

[B32] Batut B Parsons DP Fischer S Beslon G Knibbe C In silico experimental evolution: a tool to test evolutionary scenarios BMC Bioinformatics 2013 14 Suppl 1 S11 10.1186/1471-2105-14-S1-S11 24564457 PMC3851946

[B33] Ward JP Mathematical modelling of quorum sensing in bacteria Math Med Biol 2001 18 263 292 10.1093/imammb/18.3.263 11817745

[B34] Bucci V Hoover S Hellweger FL Modeling Adaptive Mutation of Enteric Bacteria in Surface Water Using Agent-Based Methods Water, Air, Soil Pollut 2011 223 2035 2049 10.1007/s11270-011-1003-6

[B35] Wang G Post WM A theoretical reassessment of microbial maintenance and implications for microbial ecology modeling FEMS Microbiol Ecol 2012 81 610 7 10.1111/j.1574-6941.2012.01389.x 22500928

[B36] Lashin SA Matushkin YG Haploid evolutionary constructor: new features and further challenges In Silico Biol 2012 11 125 35 22935966 10.3233/ISB-2012-0447

[B37] Lashin SA Suslov VV Kolchanov NA Matushkin YG Simulation of coevolution in community by using the "Evolutionary Constructor" program In Silico Biol 2007 7 261 75 18415976

[B38] Lashin SA Suslov VV Matushkin YG Comparative modeling of coevolution in communities of unicellular organisms: adaptability and biodiversity J Bioinform Comput Biol 2010 08 627 643 10.1142/S0219720010004653 20556866

[B39] Sundararaj S Guo A Habibi-Nazhad B Rouani M Stothard P Ellison M Wishart DS The CyberCell Database (CCDB): a comprehensive, self-updating, relational database to coordinate and facilitate in silico modeling of Escherichia coli Nucleic Acids Res 2004 32 Database D293 5 14681416 10.1093/nar/gkh108 PMC308842

[B40] Verhulst JH Notice sur la loi que population suit dans son accroissement Corresp mathématique Phys 1838 10 113 121

[B41] Fisher RA Average excess and average effect of a gene substitution Ann Eugen 1941 11 53 63 10.1111/j.1469-1809.1941.tb02272.x

[B42] Charnov EL Phenotypic evolution under Fisher's Fundamental Theorem of Natural Selection Heredity (Edinb) 1989 62 113 116 10.1038/hdy.1989.15 2732081

[B43] Lashin SA Mamontova EA Matushkin YG Spatially distributed modeling of prokaryotic community evolution Russ J Genet Appl Res 2013 3 184 190 10.1134/S2079059713030088

[B44] Kerr B Riley MA Feldman MW Bohannan BJM Local dispersal promotes biodiversity in a real-life game of rock-paper-scissors Nature 2002 418 171 4 10.1038/nature00823 12110887

[B45] Rubel E The Replaceability of Ecological Factors and the Law of the Minimum Ecology 1935 16 336 10.2307/1930073

[B46] Zherikhin VV Development and changes of the Cretaceous and Cenozoic faunal assemblages (Tracheata and Chelicerata) Tr Paleontol Inst Akad Nauk SSSR 1978 165 1 200

[B47] Meyen SV Fundamentals of Palaeobotany 1987 New York: Chapman & Hall 432

[B48] Darlington PJ Zoogeography: The Geographical Distribution Of Animals 1957 London: Chapman & Hall 675

[B49] Jablonski D Roy K Valentine JW Out of the tropics: evolutionary dynamics of the latitudinal diversity gradient Science 2006 314 102 6 10.1126/science.1130880 17023653

