# Supplementary material for: Modeling evolution of spatially distributed bacterial communities: a simulation with the haploid evolutionary constructor
Source: BMC Evol Biol. 2015 Feb 2;15(Suppl 1):S3. doi: 10.1186/1471-2148-15-S1-S3 (PMC4331802; doi:10.1186/1471-2148-15-S1-S3)
Supplement: Additional file 1 — Archive containing the supplementary figures. 7-Zip archive containing the supplementary figures S1-S14. [file 1471-2148-15-S1-S3-S1.zip › Figure S10.pptx]

## Slide 1
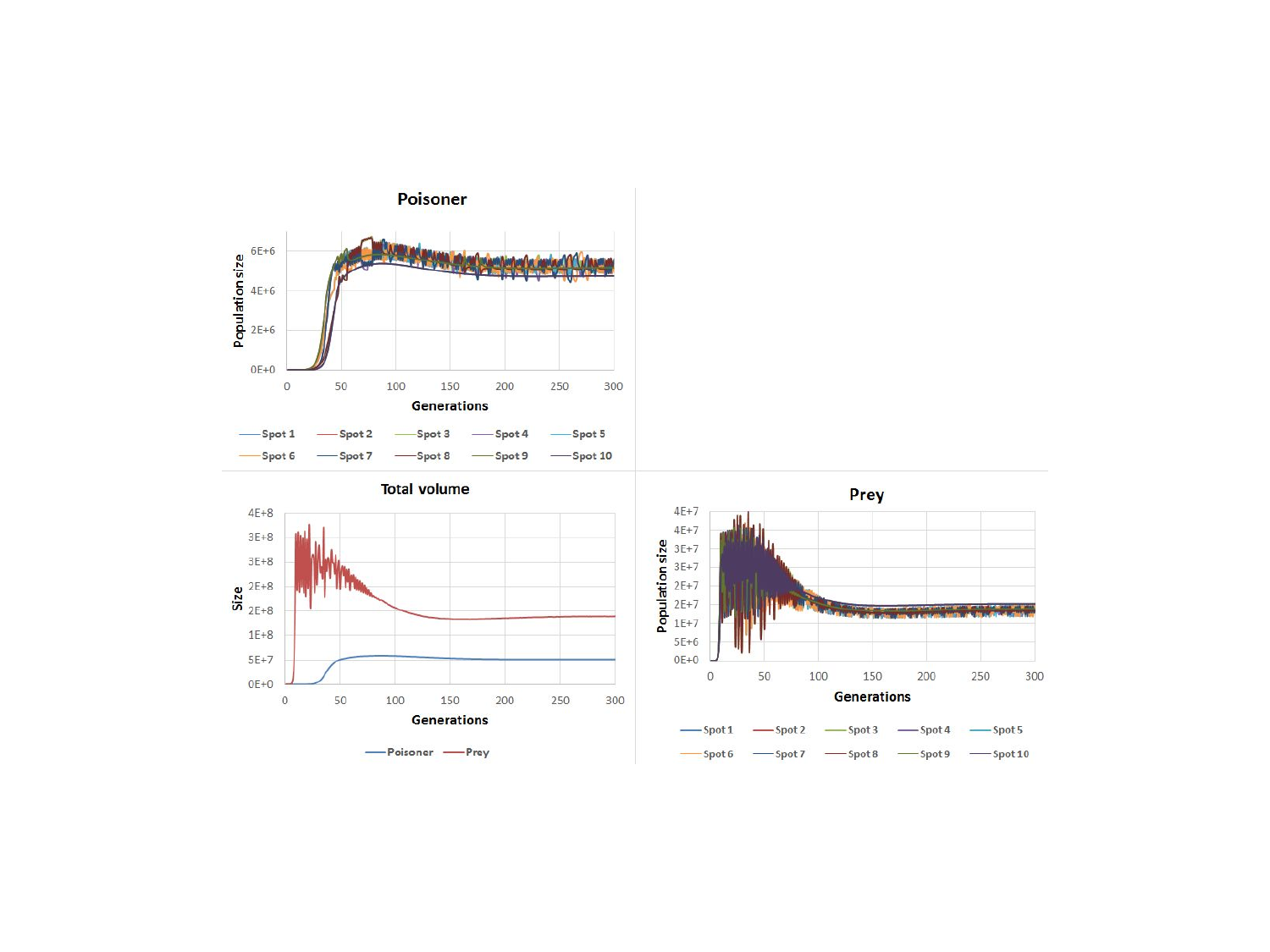

## Slide 2
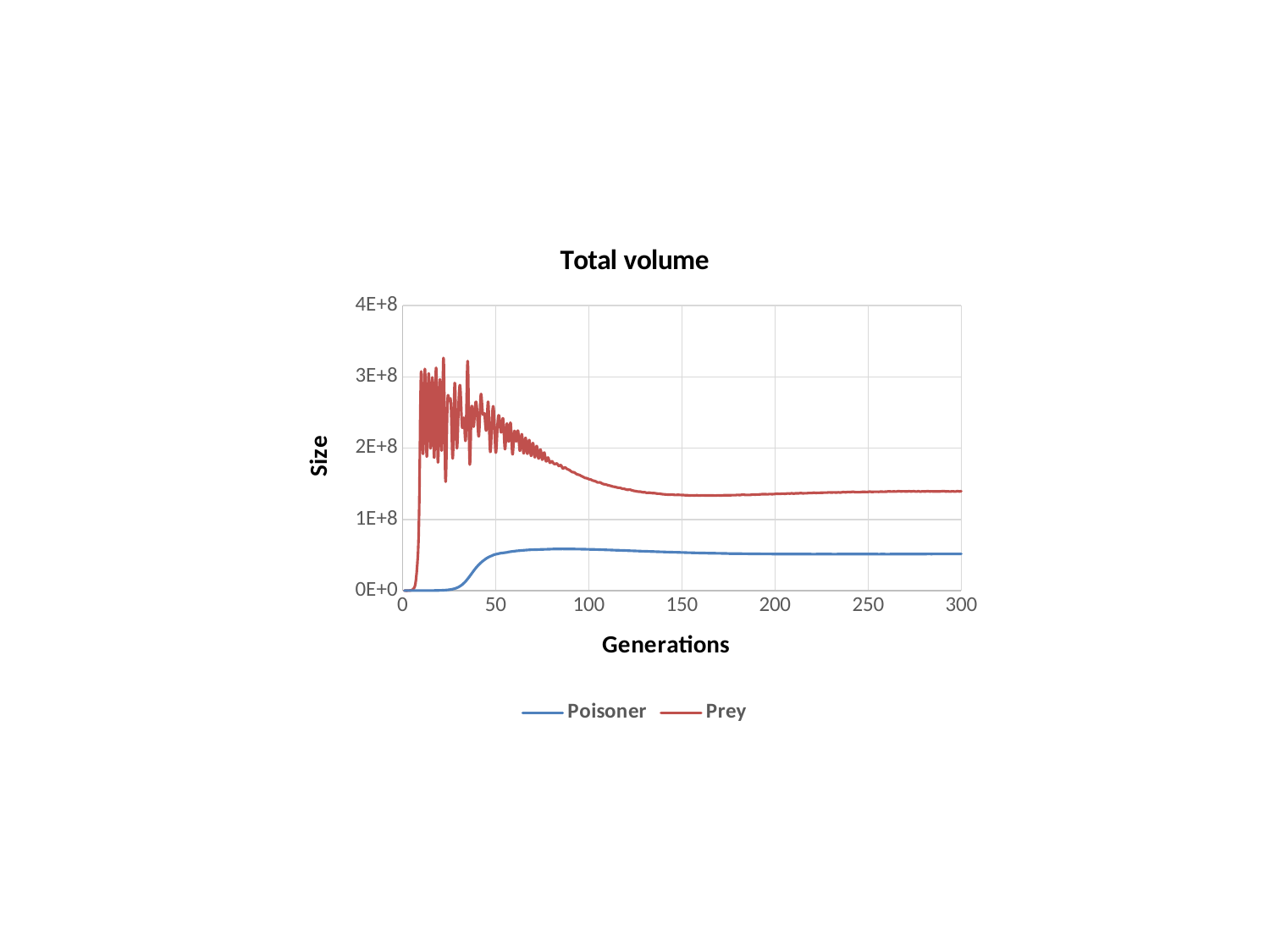

### Chart: Total volume
| Category | | |
|---|---|---|

## Slide 3
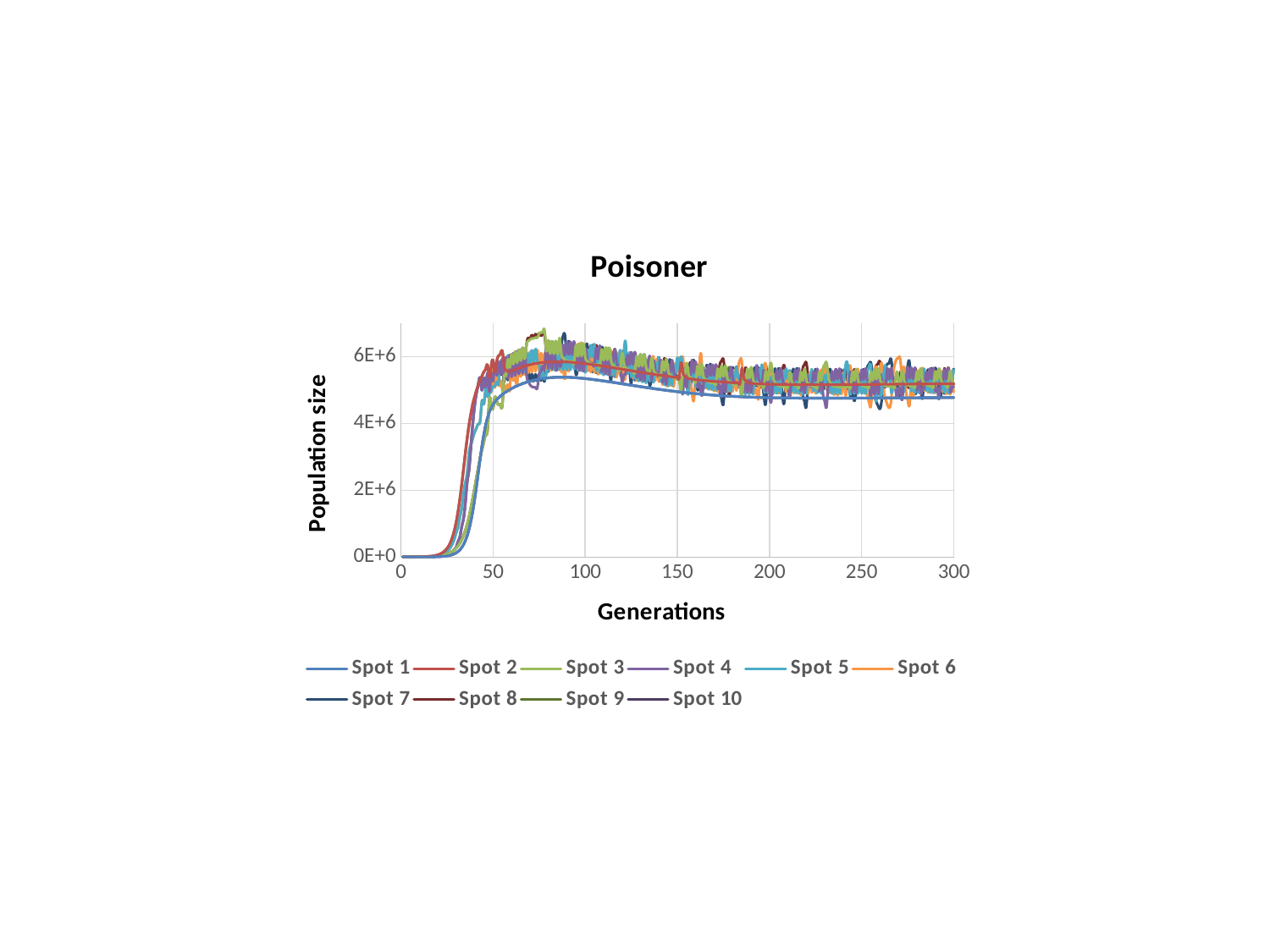

### Chart: Poisoner
| Category | Spot 1 | Spot 2 | Spot 3 | Spot 4 | Spot 5 | Spot 6 | Spot 7 | Spot 8 | Spot 9 | Spot 10 |
|---|---|---|---|---|---|---|---|---|---|---|

## Slide 4
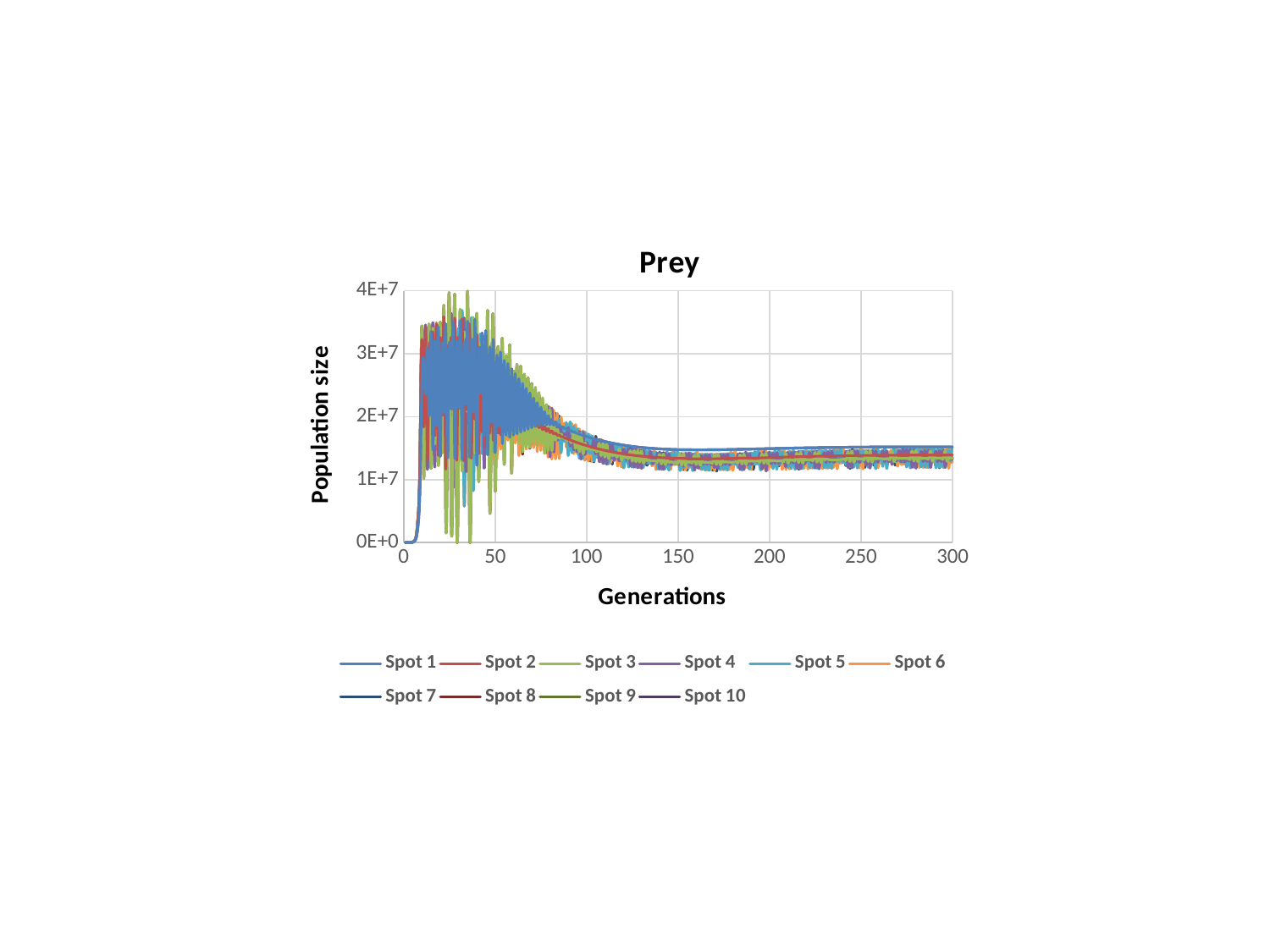

### Chart: Prey
| Category | Spot 1 | Spot 2 | Spot 3 | Spot 4 | Spot 5 | Spot 6 | Spot 7 | Spot 8 | Spot 9 | Spot 10 |
|---|---|---|---|---|---|---|---|---|---|---|
