# Supplementary material for: Modeling evolution of spatially distributed bacterial communities: a simulation with the haploid evolutionary constructor
Source: BMC Evol Biol. 2015 Feb 2;15(Suppl 1):S3. doi: 10.1186/1471-2148-15-S1-S3 (PMC4331802; doi:10.1186/1471-2148-15-S1-S3)
Supplement: Additional file 1 — Archive containing the supplementary figures. 7-Zip archive containing the supplementary figures S1-S14. [file 1471-2148-15-S1-S3-S1.zip › Figure S3.pptx]

## Slide 1
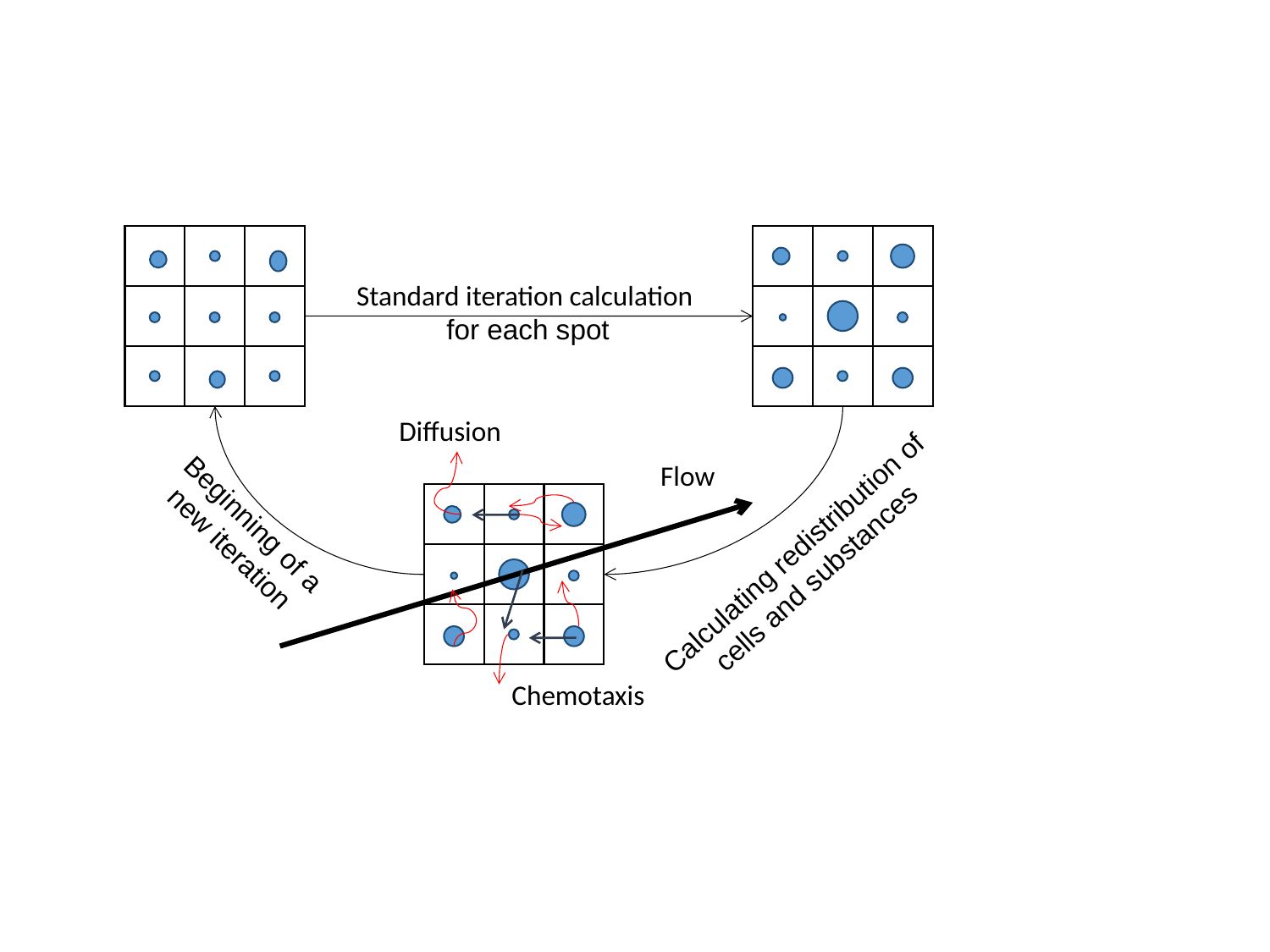

Standard iteration calculation
for each spot
Diffusion
Flow
Beginning of a new iteration
Calculating redistribution of cells and substances
Chemotaxis
