# Supplementary material for: Modeling evolution of spatially distributed bacterial communities: a simulation with the haploid evolutionary constructor
Source: BMC Evol Biol. 2015 Feb 2;15(Suppl 1):S3. doi: 10.1186/1471-2148-15-S1-S3 (PMC4331802; doi:10.1186/1471-2148-15-S1-S3)
Supplement: Additional file 1 — Archive containing the supplementary figures. 7-Zip archive containing the supplementary figures S1-S14. [file 1471-2148-15-S1-S3-S1.zip › Figure S9.pptx]

## Slide 1
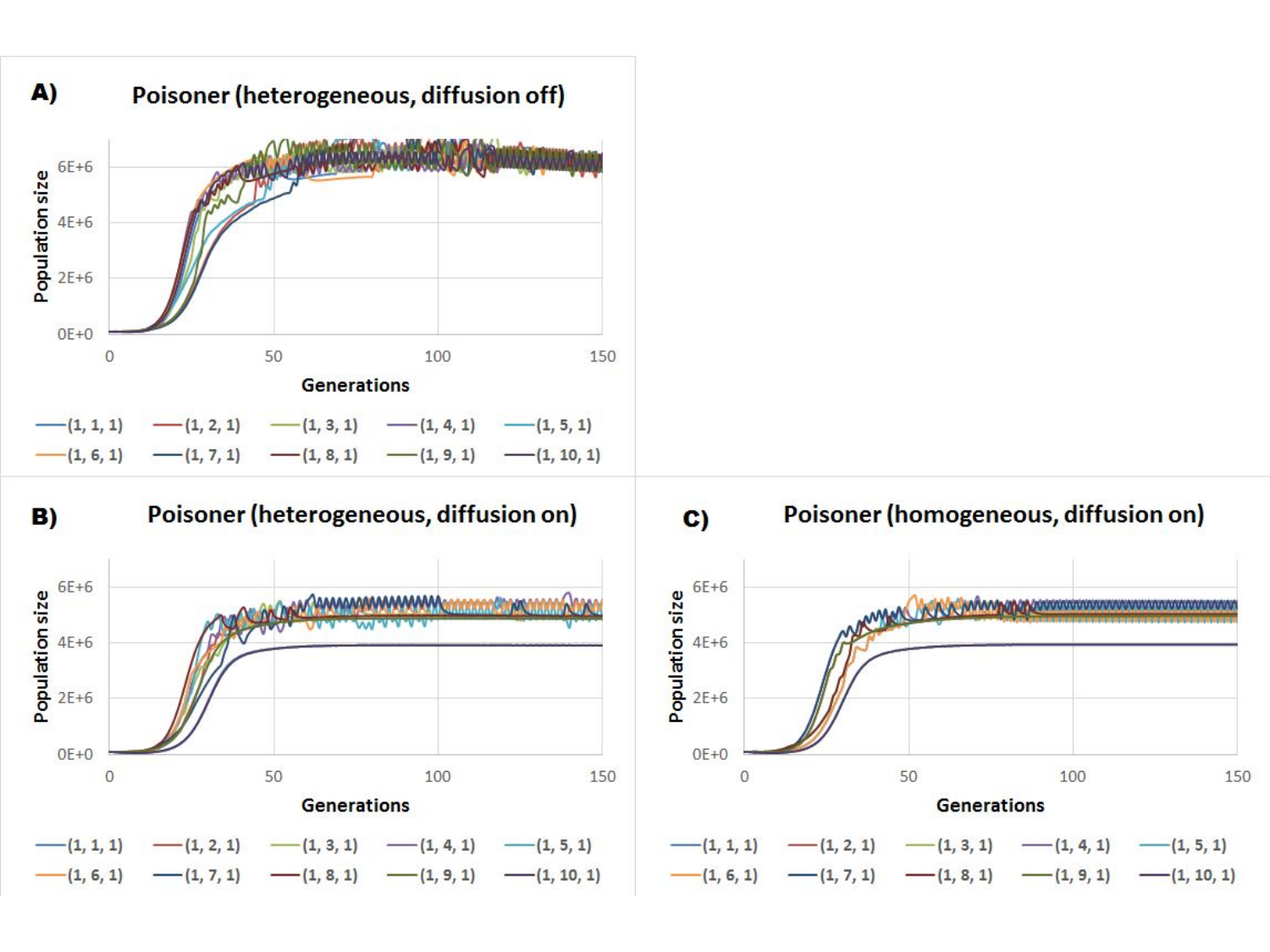

## Slide 2
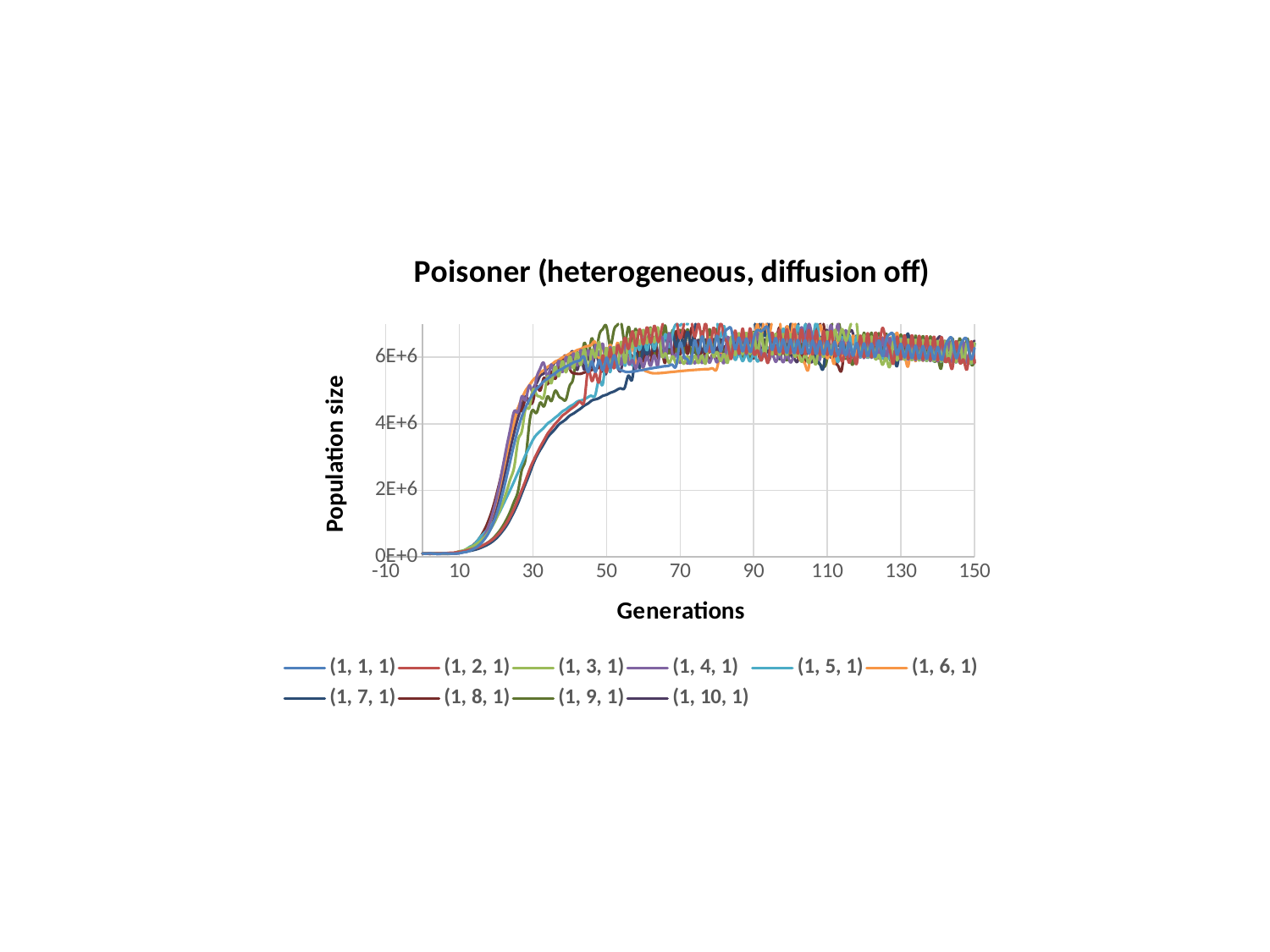

### Chart: Poisoner (heterogeneous, diffusion off)
| Category | (1, 1, 1) | (1, 2, 1) | (1, 3, 1) | (1, 4, 1) | (1, 5, 1) | (1, 6, 1) | (1, 7, 1) | (1, 8, 1) | (1, 9, 1) | (1, 10, 1) |
|---|---|---|---|---|---|---|---|---|---|---|

## Slide 3
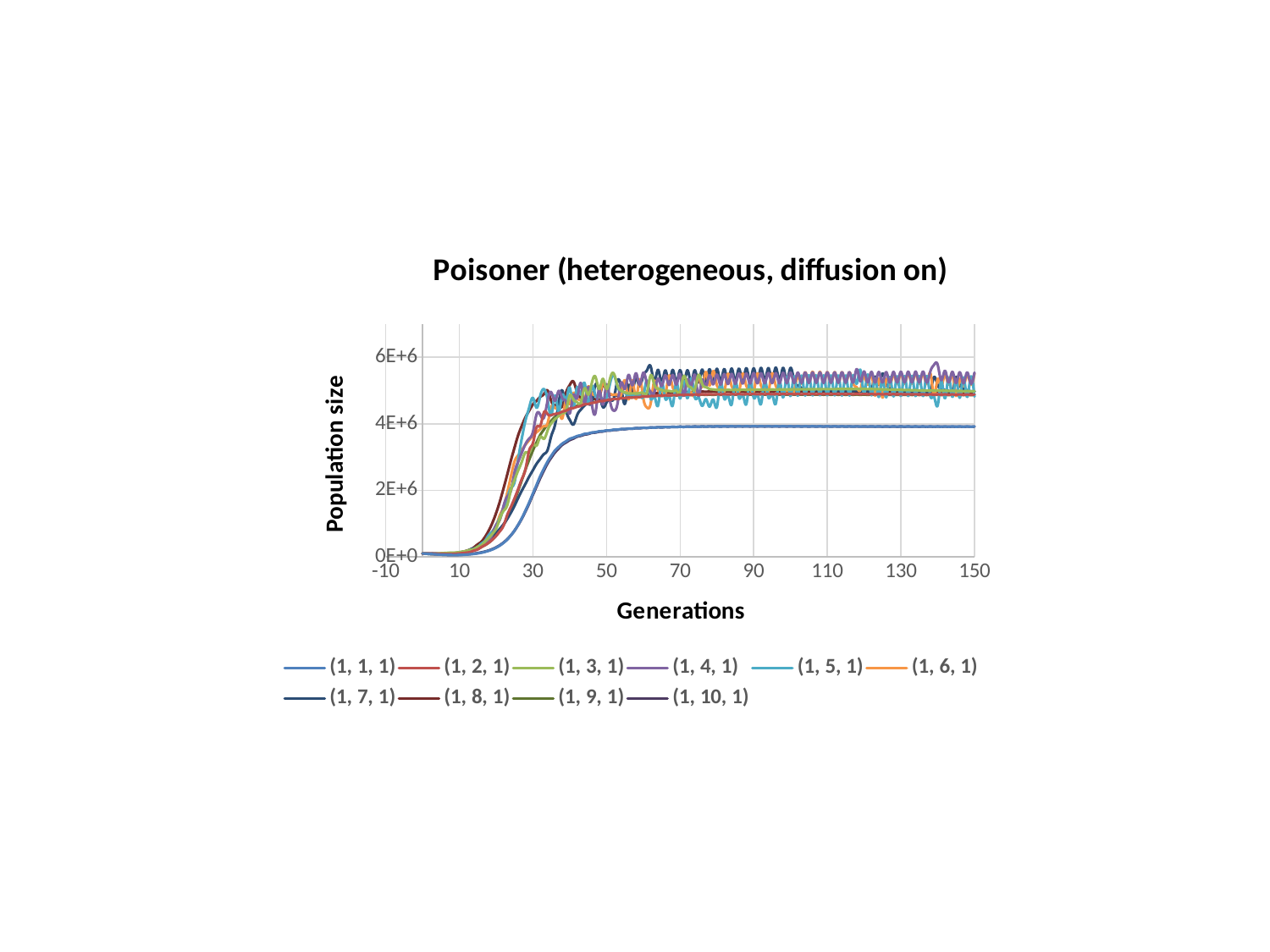

### Chart: Poisoner (heterogeneous, diffusion on)
| Category | (1, 1, 1) | (1, 2, 1) | (1, 3, 1) | (1, 4, 1) | (1, 5, 1) | (1, 6, 1) | (1, 7, 1) | (1, 8, 1) | (1, 9, 1) | (1, 10, 1) |
|---|---|---|---|---|---|---|---|---|---|---|

## Slide 4
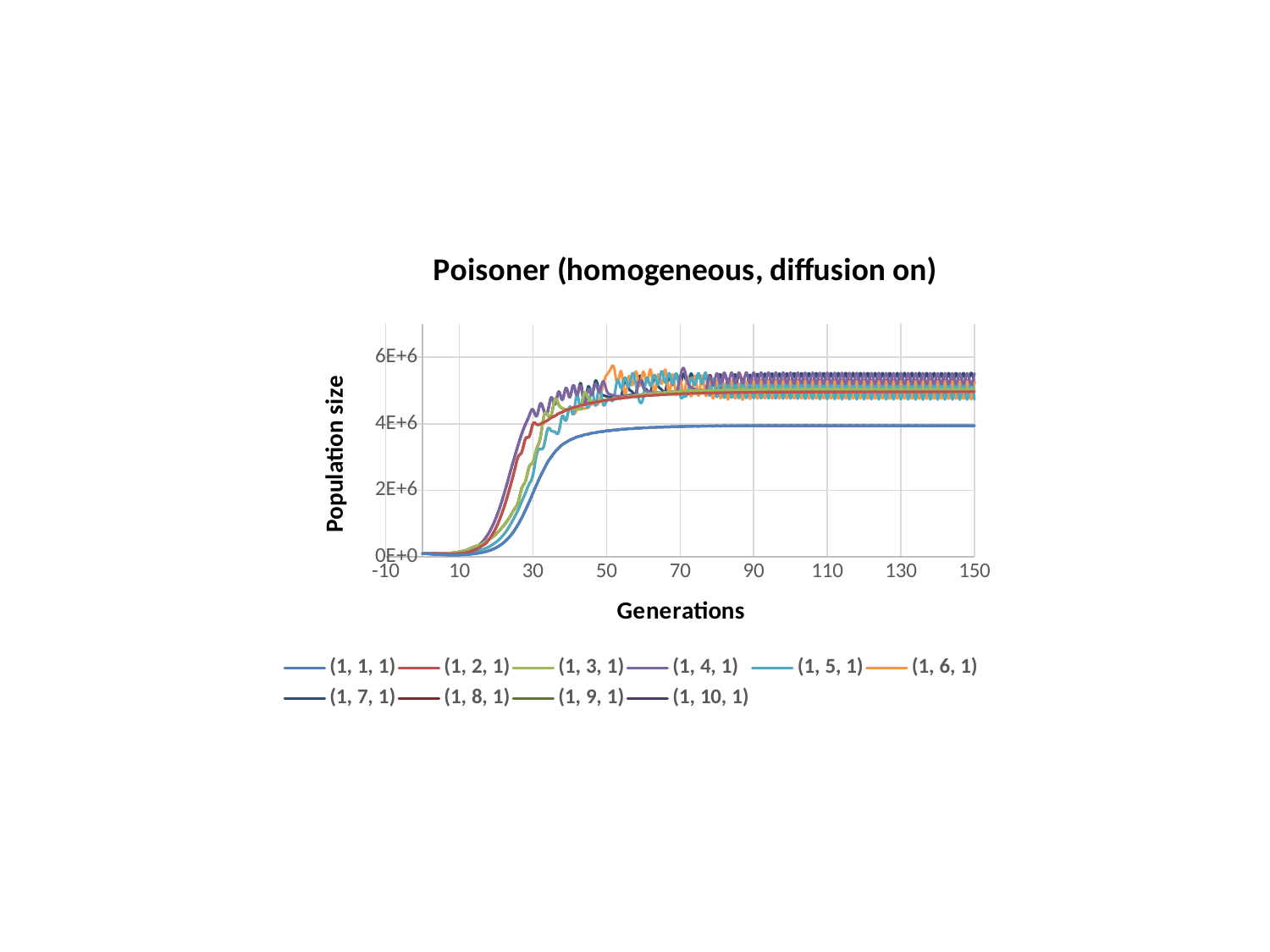

### Chart: Poisoner (homogeneous, diffusion on)
| Category | (1, 1, 1) | (1, 2, 1) | (1, 3, 1) | (1, 4, 1) | (1, 5, 1) | (1, 6, 1) | (1, 7, 1) | (1, 8, 1) | (1, 9, 1) | (1, 10, 1) |
|---|---|---|---|---|---|---|---|---|---|---|
