# Supplementary material for: Modeling evolution of spatially distributed bacterial communities: a simulation with the haploid evolutionary constructor
Source: BMC Evol Biol. 2015 Feb 2;15(Suppl 1):S3. doi: 10.1186/1471-2148-15-S1-S3 (PMC4331802; doi:10.1186/1471-2148-15-S1-S3)
Supplement: Additional file 13 — Simulation of spatially distributed habitats; Breeding strategies formulas [file 1471-2148-15-S1-S3-S13.docx]

## Simulation of spatially distributed habitats

To model spatially distributed habitats in the HEC (Additional file 14), we have added some new features. Previously, the habitat had just one spatial parameter *volume*, and no others. Cells and substances assumed to be uniformly distributed. Let us call such kind of habitat as “point (0D) environments” or “nodes”. Habitat is considered as a set of such nodes. Calculation of new states for each node (which is independent and can be performed simultaneously) includes simulation of the following processes: consumption of substrates, utilization of substrates, reproduction, substrates synthesis and secretion. This stage is apparently inherited from the HEC 0D. The additional simulation stage is redistribution of substrates and cells in the whole habitat.

Central point of the HEC extension is the simulation of the processes of transport of cells and substances between nodes. Two processes describe substrate transport: diffusion and flow. For illustrative purposes, let consider examples for 2-dimensional case. Processes in 1D and 3D are described similarly by changing dimension of vectors and connection of nodes. Let the habitat is described by a set of matrices $E_{t}^{s}=\left( \begin{matrix} e_{11} & \cdots& e_{1n} \\ \vdots& \ddots& \vdots\\ e_{m1} & \cdots& e_{mn} \end{matrix} \right)$, where $e_{ij}$- is amount of substances **s** in **(i,j)** node at iteration **t**. In order to determine spatial distribution of the substance at next step, i.e. to calculate $E_{t+1}^{s}$, we need to take into account flow and diffusion. We assume the matrix in the following way: $E_{t+1}^{s}=E_{t}^{s}+F(E_{t}^{s})+D(E_{t}^{s})$, where $F(E_{t}^{s})$ and $D\left( E_{t}^{s} \right)$ are state-transition matrices for substances (their elements might be positive and negative) in **(i,j)** node for flow and diffusion, correspondingly. Transport of cells besides diffusion and flow is determined by chemotaxis (see details below).

At each iteration step, moving of cells and substrates occurs between four neighbor nodes for 2D case for each node (2 neighbors for 1D, 6 neighbors for 3D). Therefore, the move by **n** nodes via this approach requires **n** iterations. Figure S3 shows the scheme of the iteration cycle in HEC 1D-3D.

### 1. Simulation of the flow

The flow is described by the vector of corresponding size. Vector components determine portions of cells and substances moved by the flow in each direction. By this means, a user can set the direction of flow and its intensity. Let consider the flow vector $\vec{fl}=\binom{{fl}_{x}}{{fl}_{y}}$ in Cartesian axes (in 1D and 3D cases vectors consist of one and three components, correspondingly). The sense of $\vec{fl}$ is the portion of substance removed from the node by the flow per iteration additionally normalized to obtain ${fl}_{x}+{fl}_{y}+{4k}_{d}=1$, where $k_{d}$ is normalized diffusion coefficient. It is obvious that $\left| {fl}_{x} \right|+\left| {fl}_{y} \right|\leq1$ because the amount of removed substance can not exceed the total amount. Let us denote $\vec{{fl}_{\text{abs}}}=\binom{\left| {fl}_{x} \right|}{\left| {fl}_{y} \right|}$ and let $\vec{i}=(1,0)$ and $\vec{j}=(0,1)$ are basis vectors. Let us define the matrix element $F(E_{t}^{s})=(f_{ij})$ as a dot product of two vectors: $f_{ij}=\vec{{fl}_{\text{abs}}}\cdot(c_{x}-e_{ij},c_{y}-e_{ij})$, where

$c_{x}=\left\{ \begin{aligned} e_{i,j-1}, \mathrm{if}\vec{fl}\cdot\vec{i}\geq0, \\ e_{i,j+1},\mathrm{otherwise} \end{aligned} \right.$ $c_{y}=\left\{ \begin{aligned} e_{i-1,j,}\mathrm{if}\vec{fl}\cdot\vec{j}\geq0, \\ e_{i+1,j}, otherwise \end{aligned} \right.$

### 2. Simulation of the diffusion

Diffusion is the process of indirect propagation of substance conditioned by random molecular motion in environment. In this study, the diffusion coefficient describes the portion of substance diffusing out from the current node. Change of substance amount resulted from diffusion is equal to the difference between amount of substrate came from neighbor nodes and amount of substrate removed to neighbor nodes. Therefore, diffusion of substances and cells is conditioned by difference in concentrations in neighbor nodes. To take into account the diffusion, let us consider the matrix $D(E_{t}^{s})=(d_{ij})$, where $d_{ij}=k_{d}\cdot(e_{i,j-1}+e_{i,j+1}+e_{i-1,j}+e_{i+1,j}-4e_{ij})$, $k_{d}$ is the normalized diffusion coefficient, i.e. the portion of substance diffusing from the current node into one of four neighbor nodes. Note, that we consider quadruply connected nodes and cover all in- and out-flows.

### 3. Simulation of the chemotaxis

Flow and diffusion involve both microorganisms and substances similarly. However, to adequate modeling of cells motion it is necessary to keep in view two points. First, as the most part of microbial cells might be connected to a bacterial mat, only limited portion of “free” cells might move. Second is the active motion via chemotaxis. In our method, the portion of “free” cells is described by its own coefficient, and chemotaxis is described by its own component.

Therefore, redistribution of population is described similar with substrates, but with the extended formula: $E_{t+1}^{p}=E_{t}^{p}+F(E_{t}^{p})+D(E_{t}^{p})+A(E_{t}^{p})$, where $A(E_{t}^{p})$ is the state-transition matrix for population size change in **(i,j)** node resulted from active motion of the **p** population. Each element of this matrix is calculation as the following: $a_{ij}=k_{a}*(\sum e_{uv}-w*e_{ij})$, where $k_{a}$ is the portion of free cells moved via chemotaxis, **w** is the number of neighbor nodes with favorable conditions, and $k_{a}*\sum e_{uv}$ denotes amount of cells of this species, which came from neighbor nodes with less favorable conditions.

Thus, at first we estimate attraction of environmental conditions in neighbor nodes on account of ratio between attractants and repellents in the neighbor and the current node. This way the attraction coefficients for all neighbors are calculated: $A_{pq}=\sum(b_{k}*c_{k})$, where $c_{k}$ is the concentration of **k**-th metabolite and $b_{k}= \left\{ \begin{aligned} 1, if \mathbf{k-}th metabilite is the attractant for \mathbf{p,} \\ -1, if \mathbf{k-}th metabilite is the repellent for \mathbf{p,} \\ 0, if \mathbf{p} does not consume \mathbf{k.} \end{aligned} \right.$

After that, two lists of neighbors are formed. In the first there are repellant nodes, in the second there are attractive ones. Change of population size in the current nodes is defined as the sum of the ones who came in (the first list) minus sum of the ones who went out (the second list). Moreover, the portion of actively moved cells is divided by all directions according to the normalized weight of the neighbor nodes attractions (fig. S4). It should be noted that normalizing values of $\vec{fl}$ and $k_{d}$ $k_{a}$ are also taken into account. One- and three-dimensional cases are formalized similarly. In the latter case, the $E_{t}^{s}=(e_{ijk})$ matrices are three-dimensional, and the nodes are six-connected.

## On balance between rates of reproduction and transport processes

The HEC 0D iteration cycle is assumed to correspond to the reproduction cycle. However, the addition of spatial components extends the iteration cycle by adding the transport and motion processes. Consequently, varying nodes number with keeping the habitat volume (i.e. changing physical size of nodes with keeping physical size of the whole habitat) we may obtain delay of transport processes relating to reproduction processes. It may be interpreted as the simulation of media with higher viscosity. In order to get rid of this effect, we have added the medium viscosity parameter (AV in fig. S5), which defines the number of spatial redistribution cycles per one reproduction cycle. Therefore, increasing the number of nodes in the model (keeping the physical size if the habitat), one needs to have in mind the viscosity effect.

## On Liebig’s and Rubel’s laws of ecological factors

Equations of trophic strategies are illustrated by the examples below:

$\Delta P= F_{1}\left( \vec{N},\vec{S},\vec{R},\vec{C},P \right)=\sqrt{r_{0}n\left( P \right)\cdot\sum_{i\in I_{consumed}} c_{i}s_{i}\left( P \right)}-k_{flow}\cdot P-k_{death}\cdot P^{2}$ [eq.1]

$\Delta P= F_{2}\left( \vec{N},\vec{S},\vec{R},\vec{C},P \right)=P\cdot\frac{\left( \frac{\frac{n_{0}}{P}}{K_{01\left( r_{0} \right)}} \right)^{\gamma_{0}}}{1+\left( \frac{\frac{n_{0}}{P}}{K_{02\left( r_{0} \right)}} \right)^{\gamma_{0}}}\cdot\prod_{i\in I_{consumed}} \frac{\left( \frac{\frac{s_{i}}{P}}{K_{i1\left( c_{i} \right)}} \right)^{\gamma_{i}}}{1+\left( \frac{\frac{s_{i}}{P}}{K_{i2\left( c_{i} \right)}} \right)^{\gamma_{i}}}-k_{flow}\cdot P-k_{death}\cdot P^{2}$ [eq.2]

where

*I_consumed_* – set of indices of substrates consumed;

*n_0_* – amount of nonspecific substrate, consumed by the cells of population from the environment (in proportion to the population size);

$\vec{S}$– vector of specific substrates, consumed by the cells of the population from the environment (in proportion to the population size) values;

*r_0_* – utilization rate for the unique nonspecific substrate (trait, controlled by the corresponding gene);

$\vec{C}$ – vector of corresponding specific substrate utilization rates (traits, controlled by correspondent genes);

*P* – population size;

*k_flow_* – flow rate in the environment (“washout” rate);

*k_death_* – population mortality rate;

*a_basal_* – “natural increase” of the population;

γ, γ_0_, γ_i_  – coefficients, describing the nonlinear nature of substrate influence on population growth;

*K_ij_* – coefficients, describing the efficiency of substrate influence on population growth (depends on corresponding traits).

The equation [eq.1] governs the utilization process of several specific and one nonspecific substrate, where the latter is essential for cells – when it is not available, the population does not grow. Substrates have strong cooperativity. Besides, some substrates are able to compensate in some degree the lack of other necessary substrates, including a nonspecific substrate deficiency. The trophic strategy described in [eq.1], satisfies the **Rubel’s law of replaceability of ecological factors**, and is called the **compensatory trophic strategy**.

The equation [eq.2] again governs the utilization process of several specific and one nonspecific substrate. Nevertheless, every substrate is essential. A deficiency of one substrate cannot be compensated by an excess others. The trophic strategy described by [eq.2] satisfies the ecological **Liebig’s law of the minimum** and called the **noncompensatory trophic strategy**.

## Supplementary figures (Additional file 1)

Figure S1. Main objects of HEC, their properties and relations.

Figure S2. Calculation flow in the HEC 1D-3D.

Figure S3. Principal diagram of the general iteration process for the HEC 1D-3D.

Circles of various diameters indicate microbial populations of the corresponding size. Red winding arrows indicate diffusion. Short black arrows coming out from the circles indicate migration of cells from the current node via chemotaxis. Standard HEC iteration includes utilization and production of substrates, reproduction of populations and occasionally mutations, horizontal gene transfer, and gene loss.

Fig. S4. Example of cells behavior depending on the attraction coefficients ($A_{ij}$) of the neighbor nodes (2D case).

Arrows indicate directions of the motion of cells via chemotaxis. Strikethrough arrows indicate directions of less favorable conditions.

Fig. S5. a) HEC 0D iteration cycle; b) HEC 1D-3D iteration cycle n – number of nodes, AV is the medium viscosity parameter.

Figure S6. Population dynamics in the whole habitat for the “poisoner-prey” model with the initial genetic polymorphism in both populations (Additional file 2).

Figure S7. Population dynamics of preys (top) and poisoners (bottom) in different nodes (chemotaxis is on, Additional file 15).

Figure S8. Population dynamics of preys and poisoners in the whole habitat (chemotaxis is on, Additional file 15).

Figure S9. Population dynamics of poisoners in different nodes for various model settings (chemotaxis on)

a) initially non-uniform distribution of inhibitor, diffusion is off (Additional file 15); b) initially non-uniform distribution of inhibitor, diffusion is on (Additional file 16); c) initially uniform distribution of inhibitor, diffusion is on (Additional file 17).

Figure S10. Population dynamics of poisoners and preys in the overall habitat and different nodes for the “poisoner-prey” model with the genetic polymorphism in perpendicular-flow systems.

Diffusion and chemotaxis are switched on (Additional file 18).

Figure S11. Dynamics of allele frequencies for the poisoner and prey population in nodes 1 and 73 for the “poisoner-prey” model with the genetic polymorphism in perpendicular-flow systems.

Diffusion and chemotaxis are switched on. As the spatial structure is symmetric, the same picture is observed in nodes 10 and 4, correspondingly (Additional file 18).

Figure S12. Population dynamics (C-C) in the flow-through habitat without chemotaxis (Additional file 19).

Figure S13. Population dynamics in the C-C community in the perpendicular-flow habitat.

Chemotaxis is off (left, Additional file 9) and is on (right, Additional file 10).

Figure S14. Population dynamics in the NC-NC community in the perpendicular-flow habitat.

Chemotaxis is off (left, Additional file 11) and is on (right, Additional file 12).

# Additional files

### Additional file 1

### Title: Archive containing the supplementary figures

**Description:** 7-Zip archive containing the supplementary figures S1-S14.

### Additional file 2

### Title: Archive containing the HEC script and statistic files of pp_spectre_10(through) model

**Description:** 7-Zip archive containing text file with the model script and statistic files concerned the results depicted in figures 5-6, S6, table 1.

### Additional file 3

### Title: Archive containing the HEC script and statistic files of ort_10_d=0 model

**Description:** 7-Zip archive containing text file with the model script and statistic files concerned the results depicted in figure 7.

### Additional file 4

### Title: Archive containing the HEC script and statistic files of Reissue.Rubel.through.chem=0.1.hgt1 model

**Description:** 7-Zip archive containing text file with the model script and statistic files concerned the results depicted in figure 9 (left side).

### Additional file 5

### Title: Archive containing the HEC script and statistic files of Reissue.Rubel.through.chem=0.1.hgt10 model

**Description:** 7-Zip archive containing text file with the model script and statistic files concerned the results depicted in figure 9 (right side).

### Additional file 6

### Title: Archive containing the HEC script and statistic files of Reissue.Liebig.through.chem=0.1.hgt1 model

**Description:** 7-Zip archive containing text file with the model script and statistic files concerned the results depicted in figure 10.

### Additional file 7

### Title: Archive containing the HEC script and statistic files of Reissue.Liebig.through.chem=0.1.hgt5 model

**Description:** 7-Zip archive containing text file with the model script and statistic files concerned the results depicted in figure 10.

### Additional file 8

### Title: Archive containing the HEC script and statistic files of Reissue.Liebig.through.chem=0.1.hgt10 model

**Description:** 7-Zip archive containing text file with the model script and statistic files concerned the results depicted in figure 10.

### Additional file 9

### Title: Archive containing the HEC script and statistic files of Reissue.Rubel.ort model

**Description:** 7-Zip archive containing text file with the model script and statistic files concerned the results depicted in figure S13 (left side).

### Additional file 10

### Title: Archive containing the HEC script and statistic files of Reissue.Rubel.ort .chem=0.1 model

**Description:** 7-Zip archive containing text file with the model script and statistic files concerned the results depicted in figure S13 (right side).

### Additional file 11

### Title: Archive containing the HEC script and statistic files of Reissue.Liebig.ort model

**Description:** 7-Zip archive containing text file with the model script and statistic files concerned the results depicted in figure S14 (left side).

### Additional file 12

### Title: Archive containing the HEC script and statistic files of Reissue.Liebig.ort .chem=0.1 model

**Description:** 7-Zip archive containing text file with the model script and statistic files concerned the results depicted in figure S14 (right side).

**Additional file 13**

**Title: Simulation of spatially distributed habitats**

### Additional file 14

### Title: Archive containing the HEC executable file

**Description:** 7-Zip archive containing the HEC executable file (Windows version).

### Additional file 15

### Title: Archive containing the HEC script and statistic files of ort_10_d=0 model

**Description:** 7-Zip archive containing text file with the model script and statistic files concerned the results depicted in figures S7-S8, S9a.

### Additional file 16

### Title: Archive containing the HEC script and statistic files of ort_10_d=0.01 model

**Description:** 7-Zip archive containing text file with the model script and statistic files concerned the results depicted in figure S9b.

### Additional file 17

### Title: Archive containing the HEC script and statistic files of ort_10_d=0.01(homog) model

**Description:** 7-Zip archive containing text file with the model script and statistic files concerned the results depicted in figure S9c.

### Additional file 18

### Title: Archive containing the HEC script and statistic files of pp_spectre_10(ort) model

**Description:** 7-Zip archive containing text file with the model script and statistic files concerned the results depicted in figure S10-S11.

### Additional file 19

### Title: Archive containing the HEC script and statistic files of Reissue.Rubel.through model

**Description:** 7-Zip archive containing text file with the model script and statistic files concerned the results depicted in figure S12.
